# Performance Comparison of Recombinant Baculovirus and Rabies Virus-like Particles production Using Two Culture Platforms

**DOI:** 10.3390/vaccines11010039

**Published:** 2022-12-24

**Authors:** Luis Giovani Oliveira Guardalini, Paulo Eduardo da Silva Cavalcante, Jaci Leme, Renata Gois de Mello, Thaissa Consoni Bernardino, Simone Gonçalves Silva Jared, Marta Maria Antoniazzi, Renato Mancini Astray, Aldo Tonso, Eutimio Gustavo Fernández Núñez, Soraia Attie Calil Jorge

**Affiliations:** 1 Laboratório de Biotecnologia Viral, Instituto Butantan, Av Vital Brasil 1500, São Paulo CEP 05503-900, SP, Brazil; 2 Laboratório de Biologia Estrutural, Instituto Butantan, Av Vital Brasil 1500, São Paulo CEP 05503-900, SP, Brazil; 3 Laboratório Multipropósito, Instituto Butantan, Av. Vital Brasil 1500, São Paulo CEP 05503-900, SP, Brazil; 4 Laboratório de Células Animais, Departamento de Engenharia Química, Escola Politécnica, Universidade de São Paulo. Av. Prof. Luciano Gualberto, Trav. 3, 380, São Paulo CEP 05508-900, SP, Brazil; 5 Grupo de Engenharia de Bioprocessos, Escola de Artes, Ciências e Humanidades (EACH), Universidade de São Paulo, Rua Arlindo Béttio, 1000, São Paulo CEP 03828-000, SP, Brazil

**Keywords:** rabies virus-like particles, recombinant baculovirus, Sf9 cells, stirred-tank bioreactor, viral infection, quality by design

## Abstract

This work aimed to assess, following upstream optimization in Schott flasks, the scalability from this culture platform to a stirred-tank bioreactor in order to yield rabies-recombinant baculovirus, bearing genes of G (BVG) and M (BVM) proteins, and to obtain rabies virus-like particles (VLP) from them, using Sf9 insect cells as a host. Equivalent assays in Schott flasks and a bioreactor were performed to compare both systems and a multivariate statistical approach was also carried out to maximize VLP production as a function of BVG and BVM’s multiplicity of infection (MOI) and harvest time (HT). Viable cell density, cell viability, virus titer, BVG and BVM quantification by dot-blot, and BVG quantification by Enzyme-Linked Immunosorbent Assay (ELISA) were monitored throughout the assays. Furthermore, transmission electron microscopy was used to characterize rabies VLP. The optimal combination for maximum VLP expression was BVG and BVM MOI of 2.3 pfu/cell and 5.1 pfu/cell, respectively, and 108 h of harvest time. The current study confirmed that the utilization of Schott flasks and a benchtop bioreactor under the conditions applied herein are equivalent regarding the cell death kinetics corresponding to the recombinant baculovirus infection process in Sf9 cells. According to the results, the hydrodynamic and chemical differences in both systems seem to greatly affect the virus and VLP integrity after release.

## 1. Introduction

Rabies is one of the oldest known zoonotic diseases. It is triggered by a negative-stranded RNA virus from the *Lyssavirus genus*. The rabies genome consists of approximately 12 kb that encodes five structural proteins: the nucleoprotein (N), phosphoprotein (P), matrix protein (M), glycoprotein (G), and RNA-dependent RNA polymerase (L) [1]. The illness is still considered a public health problem, especially in low- and middle-income countries, killing about 59,000 people worldwide per year (95 % in Asia and Africa) and costing USD 8.6 billion in economic losses [2,3]. Without prophylaxis, its fatality rate is close to 100%. Safe and effective vaccines are commercially available for use both as pre- and post-prophylaxis [3].

The human rabies vaccines are manufactured from several culture systems and seed viruses, and all are required to achieve a potency of 2.5 IU per intramuscular dose. The culture systems commonly used are nerve tissue, avian embryos, and cell cultures. After the virus’s propagation in the system, the virus is inactivated using mainly phenol, β-propriolactone, and formalin. Despite modern cell culture vaccines having improved the safety and effectiveness of human rabies vaccines, they continue to be expensive (due to their scarce availability in rural clinics, the requirement for several doses and refrigeration, etc.). Moreover, few nerve tissue vaccines, which have adverse reactions, are still produced. To overcome these barriers, a new generation of rabies vaccines is underway. These novel platforms include adjuvanted human rabies vaccines, nanoparticle vaccine technology, adenovirus vector with E1 deletion or chimpanzee origin expressing the rabies G-protein, and virus-like particles (VLP) [4,5].

VLP are a kind of subunit vaccine based on structural viral proteins, which are assembled to make up a particle. The major advantages of VLP are their morphological similarity to the native virus, highly repetitive immunogenic surface structure, and the conservation of cell uptake and immune processing pathways associated with their parent virus. In addition, the nonpathogenic nature of VLPs significantly improves their safety. There are a set of commercially available VLP vaccines against the hepatitis B virus, hepatitis E virus, and human papillomavirus, and another broad group of vaccines is under investigation [6]. Several VLP production platforms such as bacteria, yeast, insect cells, mammalian cell, and plants have been used. Specifically, the baculovirus-insect cell expression system (BVES) is one of the most widely utilized for the production of VLP. The main hosts of BVES are the insect cell lines derived from *Spodoptera frugiperda* and *Trichoplusia ni*, which grow at around 28 °C and do not require CO_2_. These attributes make the scale-up of protein production feasible. Nevertheless, the key limitations of the BVES are its low yield of secreted and membrane-bound proteins as well as the isolation of the VLP from numerous impurities [7].

The robustness of insect cell lines mentioned above in terms of pH is also extended to dissolved oxygen tension [8]. In previous studies, these features have allowed for the demonstration of similar results in non-well-controlled culture systems, such as those provided by Schott flasks and wave bioreactors, concerning heterologous protein expression in recombinant constitutive insect cell lines [9]. However, other scientific communications have reported different performances, especially in stirred-tank bioreactors. Yet, for optimizing expression system conditions at low cost in insect cell lines, the Schott flask system has been used repeatedly [10,11]. To date, reports regarding the upstream process optimization of recombinant baculovirus and VLP using Schott flasks are scarce. The key parameters—concerning this process in batch operation mode—still to be defined are the multiplicity of infection (MOI), time of infection (TOI), and harvest time (HT) [12].

On the other hand, the quality by design (QbD) concept has been applied in the biopharmaceutical industry. QbD has an important role in guiding and improving a product’s design and production process and helping to decrease development expenses. One of the crucial elements of QbD is the Design of experiments (DoE), which enables the creation of a design space and is used in optimization studies to define the effects of interactions of multiple factors at a time. The DoE is also a useful tool for experimental planning to obtain data, which may be analyzed to obtain suitable objective conclusions [13].

This work aimed to identify, following upstream DoE optimization in Schott flasks, the similarities, and differences between this culture system and the bench-stirrer tank bioreactor. The rabies recombinant baculovirus, which bears protein G (BVG) and M (BVM), as well as the rabies VLP, obtained from them and using Sf9 insect cells as a host, were analyzed. The findings from this study can guide equivalent platform developments for vaccines’ upstream stage, making scale-up more affordable in terms of resources and time.

## 2. Materials and Methods

### 2.1. Cell Lines and Culture Media 

Sf9 cells in suspension (ATCC 1711) cultivated in serum-free SF900III medium (Gibco, Thermo Fisher Scientific, Waltham, MA, USA) were chosen for baculovirus propagation and VLP production. In addition, the Sf9 ET (easy titration) cells cultivated in supplemented SF900III medium with 2.5% (*v*/*v*) fetal bovine serum (FBS, HyClone^®^, Cytiva, Marlborough, MA, USA) were used in viral titration assays, which were kindly provided by professors Ralph Hopkins and Dominic Esposito from the National Cancer Institute at Frederick (MD, USA) [14]. Moreover, Sf9 cells were also cultivated in a monolayer over the surface of 25 cm^2^ T-flask (Corning Inc.™, Corning, New York, NY, USA) for cell transfection assays.

### 2.2. Viral Stock and Conservation

Viral batches were obtained by transfection of 10 µg of recombinant RVGP and RVM bacmid complexed with cationic liposome (Cellfectin II^®^, Invitrogen, Carlsbad, CA, USA) separately in 5 × 10^6^ Sf9 cells, using a 25 cm^2^ T-flask in 5 mL of SF900III medium, and culturing them for 96 h. The genetic constructions were defined and implemented as described previously [15]. Then, the supernatant was clarified and stored at 4 °C and protected from light, giving rise to passage batch 1. To increase the number of infective particles used in the infection and co-infection assays, two consecutive infections were performed in Schott flasks (with a total volume of 100 mL and a working volume of 20 mL), with a multiplicity of infection of 0.1 pfu/cell at a cell density of 1 × 10^6^ cells/mL and employing a 100 rpm rotation speed for 96 h. The culture supernatant was clarified and stored at 4 °C and protected from light, giving rise to passage batches 2 and 3, with the latter being used for the infection and co-infection tests.

### 2.3. Cell Inoculum Preparation and General Conditions for Assays in Schott Flasks

To perform all assays in Schott flasks and to prepare bioreactor inocula, three passages were done after thawing cell vials from a working bank (totaling less than 20 passages after thawing the vials with the cells provided by the ATCC). The initial cell density was within the 0.5–1 × 10^6^ cells/mL range. The cultures were performed in 100 mL shake flasks (Schott AG™, Mainz, Germany) with a working volume of 20 mL. The rabies VLP yield optimization, as well as viral production and titration of batches, were performed via orbital shaking at 100 rpm, whereas 120 rpm was used for experimental runs, which aimed to compare the production of recombinant baculoviruses BVG and BVM to those obtained in a bioreactor. All cultures using Schott flasks were performed in a rotatory incubator shaker (Innova 4000, New Brunswick Scientific, Edson, New Jersey, NJ, USA).

### 2.4. Optimization and Confirmation Assays in Schott Flasks

The rabies VLP production optimization assays were performed using multiplicities of infection (MOIs), as described in Table 1, for hosts infected with a cell concentration of 1 × 10^6^ cells/mL and maintained for 120 h. Samples were collected daily to define cell density (Xv), cell viability, and immunochemical quantifications.

To compare the performance between both culture systems, the production of recombinant baculoviruses BVG and BVM, as well as rabies VLP, were assessed in Schott flasks and a bioreactor under similar conditions. The experimental runs were performed at an initial cell density of 6–7 × 10^5^ cells/mL. Infection of each BV, with an MOI of 0.1 pfu/cell, was performed after one day of cell inoculum (TOI of 24 h), at which time the viable cell concentration was around 1.3–1.6 × 10^6^ cells/mL. The VLP production by co-infection of BVG and BVM also simulated the same conditions of the assay carried out in the bioreactor.

### 2.5. Bioreactor Assays

The bioreactor runs were carried out in a BioFlo^®^ 110 (New Brunswick Scientific, Edison, New Jersey, NJ, USA) apparatus with 2 L of nominal vessel volume and 1 L working volume, coupled with a computer with homemade software implemented in the LabVIEW programming language software (National Instruments, Austin, TX, USA), which records the variables pH, temperature, agitation speed, and dissolved oxygen in the medium (DO) in real-time. To determine Xv and viability, viral titer, and immunochemical parameters, and obtain micrographs, up to three daily samples were removed. For these assays in the bioreactor, the parameters defined were as follows: a specific flow rate equal to 0.2 volume of gas per volume of culture medium per minute (vvm), dissolved oxygen tension in the medium at 30% of saturation with air, a temperature of 28 °C, and stirrer speed of 80 rpm. The cell inoculum density of the assays was between 5–8 × 10^5^ cells/mL, and the infection (BVG and BVM MOIs of 0,1 pfu/cell) or co-infection (BVG and BVM MOIs of 3 and 2 pfu/cell, respectively) occurred at TOI around 24 h.

### 2.6. Cell Density and Viability

The viable cell concentration (Xv) was measured by counting the cells in 8 quadrants of the Neubauer chamber using a light microscope. The cell viability quantification was performed through the dye exclusion method using Trypan Blue 0.04% (*w*/*v*) [16].

### 2.7. Viral Titration

For viral titration assay, Sf9 ET cells were seeded in a 96-well plate at a concentration of 8 × 10^4^ cells/well in 180µL of SF900III without fetal bovine serum supplementation. The viral lot to be titrated was serially diluted from 10^−1^ to 10^−8^ in SF900III medium and 20 µL of the dilutions was applied to the plate in octuplicates, following an established protocol [17]. After 96 h, the supernatant was exchanged for Phosphate-Buffered Saline (PBS, 1.76 mM KH_2_PO_4_, 130 mM NaCl, 2.68 mM KCl, 10.14 mM Na_2_HPO_4_–pH 7.0). Then, the plate was observed using an inverted fluorescence microscope to count fluorescence spots; then, viral titer calculation was performed. Sf9 ET cells have the enhanced green fluorescent protein (eGFP) gene transfected under the polyhedrin promoter. Thus, when cells are infected by baculoviruses, they end up activating this promoter, causing the infected cells to fluoresce.

### 2.8. Dot Blotting

The primary mouse anti-rabies glycoprotein antibody (LifeSpan BioSciences, LSBio–C75309, Seattle, WA, USA), at a dilution of 1:4000, and the primary rabbit anti-rabies matrix antibody (Cusabio Biotech, CSB-PA322192LA01RAI, Texas, TX, USA), at a dilution of 1:2000 dilution, were used to accomplish the dot-blotting assays. As secondary antibodies, the goat HRP anti-mouse antibody (Thermo Fisher Scientific, G21040, Waltham, MA, USA), at a dilution of 1:4000, and the goat HRP anti-rabbit antibody (Thermo Fisher Scientific, 656120, Waltham, MA, USA), at a dilution of 1:5000, were used. Briefly, 100 µL of the sample was applied to a nitrocellulose membrane (GE Healthcare Life Science, Chicago, IL, USA) in the Bio-Dot™ Apparatus device (BioRad, São Paulo, Brazil) and incubated for 90 min in blocking solution (3% *w*/*v* milk skimmed powder in PBS 1X). Then, the membrane was washed with PBS 1X+ 0.05% Tween 20 three times, with each wash performed for 10 min. Afterward, the membrane was incubated for 1 h in the corresponding primary antibody and washed three times with PBS 1X + 0.05% Tween 20. The membrane was immediately incubated with the corresponding secondary antibody for 1 h and washed three times with PBS 1X + 0.05% Tween 20, with 10 min each wash. The signal exposure was achieved using the PicoWest SuperSignal Chemiluminescent Substrate kit (Thermo Fisher Scientific, Waltham, MA, USA), following the manufacturer’s recommendations. The images were recorded in the Alliance 2.7 Photodocumenter (Uvitec, Cambridge, UK).

Spots’ optical densities from dot-blot membranes were measured using the Uviband Max software version 1506 (UVITEC Cambridge^®^, Cambridge, UK). The optical densities were transformed into intensity signals by the software. With these numbers, it was possible to correlate the intensities of the samples with the intensities of the standard curve applied on the membrane through linear regression. For the standard calibration curve (125–2000 ng/mL), previously quantified inactivated rabies virus was used.

### 2.9. Enzyme-Linked Immunosorbent Assay (ELISA) for RVGP

A Rabies Glycoprotein Enzyme Immunoassay kit (Pasteur Institute, Paris, France), which has been described previously [18], was used to quantify RVGP via ELISA. Briefly, a 96-well plate was sensitized with 200 µL of Mab-D1 primary antibody, diluted at a ratio of 1:2000 in carbonate buffer (50 mM NaHCO_3_ solution buffered with 50 mM Na_2_CO_3_ solution) with pH 9.6, and incubated for 16 h at 4 °C. Then, the plate was blocked with 300 µL of the blocking solution (5% sucrose *w*/*v* and 3% BSA *w*/*v*, in carbonate buffer) and incubated for 30 min at 37 °C. Afterward, the plate was washed five times with buffer 1X PBS + 0.05% Tween (washing buffer), and 150 µL of the standard curve (inactivated and lyophilized rabies virus, present in the kit) or sample supernatant was added in duplicate. Then, the plate was incubated for one hour at 37 °C. Subsequently, the plate was washed six times with washing buffer and 200 µL of peroxidase-conjugated primary antibody MAb-D1-Po (Pasteur Institute, Paris, France) diluted 1:2000 in conjugate buffer (1% BSA *m*/*v*, 0.05% Tween 20 *v*/*v*, in 1X PBS, pH 7.0) was applied to the plate, which was incubated for an additional hour at 37 °C. The plate was washed again six times in washing buffer and 200 µL of chromogen substrate (2 mg/mL orthophenylenediamine, 1 µL/mL H_2_O_2_, in citrate buffer–45 mM sodium citrate, 16.3 mM citric acid anhydrous, pH 5.6) was applied to the plate, which was incubated for 30 min at room temperature and protected from light. Finally, 50 µL of stop solution (4N Sulfuric Acid) was added. The absorbance in each plate well was measured in a plate reader (Multskan EX, Thermo Scientific, Waltham, MA, USA) at a wavelength of 492 nm.

### 2.10. Western Blots for Detection of G and M proteins

Western-blotting procedures began with protein separation from the infected culture supernatant samples via SDS-PAGE electrophoresis in 10% polyacrylamide gel. The current used was 0.03 A per gel with constant amperage. Samples were loaded in the running buffer NuPage LDS Sample Buffer 1X (Thermo Fisher Scientific, Waltham, MA, USA). The inactivated rabies virus from the Pasteur Institute (Paris, France) was used as a positive control (at a concentration of 125–2000 ng/mL). The RulerPlus Ruler II marker (Transbionovo, Beijing, China) was used as a reference to identify the proteins’ molecular weights. After the run, the gel was transferred to the nitrocellulose membrane (GE HealthCare Life Science) in the Mighty Small Tank Transfer Unit apparatus (Amersham-GE, Chicago, IL, USA), immersed in transfer buffer (38 mM glycine, 0.1% (*w*/*v*) of SDS and 48 mM of Tris, and 20% ethanol (*v*/*v*)) for 16 h with a constant voltage of 30 Then, the transferred nitrocellulose membrane was processed following the same steps described in the dot-blotting procedure.

### 2.11. Transmission Electron Microscopy

In order to characterize rabies virus-like particles, transmission electron microscopy (TEM) was performed using the Zeiss Leo 906 E device (Electron Microscopy, Düsseldorf, Germany). Initially, the samples were concentrated at 150,000 g for 90 min. The pellet formed was resuspended in PBS 1X and passed through a sucrose cushion (20% *w*/*v*) at 230,000 g for 2 h. Then, the new pellet was resuspended in PBS 1X; 10 µL of the sample was applied to copper grids pre-coated with parlodium and incubated for 10 min at room temperature. Subsequently, 5 µL of an aqueous solution of 2% (*w*/*v*) uranyl acetate was added and incubated for 1 min. After drying, micrographs of the grids were taken on the electron microscope. Regarding the immunostaining of the samples, the primary mouse anti-rabies glycoprotein antibody (LifeSpan BioSciences, LSBio–C75309, Seattle, WA, USA) was used, at a dilution of 1:4000, as well as the primary rabbit anti-rabies matrix antibody (Cusabio Biotech, CSB-PA322192LA01RAI, Texas, TX, USA), at a dilution of 1:2000. The secondary antibodies used, were goat anti-mouse antibody conjugated with 10 nm of colloidal gold (ThermoFisher Scientific, A31561, Waltham, MA, USA) and goat anti-rabbit antibody conjugated with 5nm of colloidal gold (ThermoFisher Scientific, A31565, Waltham, MA, USA), which were both diluted at a proportion of 1:100. The samples were placed in copper grids coated with parlodium and incubated for 15 min. After that, they were incubated for 1 h with the corresponding primary antibody in a dark and humid environment. Then, the grids were washed 4 times with 1X PBS + 1% BSA, with 10 min taken for each wash. Again, they were incubated for 1 h in a humid and dark environment with the corresponding secondary antibody. After that, the grids were washed four times with saline solution (0.9% NaCl). Finally, the grids were dripped with 10 µL of 2% uranyl acetate (*w*/*v*) and read after drying.

### 2.12. Data Analysis

The comparisons among different treatments were performed using one-way ANOVA with the aid of StatGraphics^®^ Centurion version 19 software (Statgraphics Technologies, Inc., Virginia, VA, USA). In treatments that showed a statistically significant difference determined via one-way ANOVA (*p*-value < 0.05), Tukey-type multiple comparison tests were performed. The design, data modeling, and optimization of the statistical experiment were performed using Design Expert^®^ version 13 software (Stat-Ease, Inc., Minneapolis, MN, USA). For the co-infection tests, VLP production optimization was carried out using the desirability function according to the criteria described in Table 2. The used desirability function method can solve the simultaneous optimization of several response variables using univariate techniques [19,20]. To demonstrate the similarity among the values of the response variables predicted by statistical modeling and those determined empirically (three repetitions) in Schott flasks using the same operating conditions, a hypotheses test was performed using a significance level equal to 0.5 (α = 0.5).

The maximum specific growth rate $\left(\mu_{\max}\right)$ was calculated as the slope from the first-order Equation fitted to $\mathrm{Ln}\left(X_{v}\right)-t\;$data over the exponential growth phase. The cell-doubling time was determined by the equation $\frac{\ln\left(2\right)}{\mu_{\max}}$. The instant cell death rate after BV infections was defined as the slope of the first-order equation fitted to $X_{v}-t$ data over the post-infection phase.

## 3. Results and Discussion

The use of low-cost, monitorable, and scalable approaches for develop and optimize the upstream stage of biopharmaceutical protein production, which makes extensive use of animal cells, mainly mammalian cell lines, is still a challenge. This can be accomplished to some extent for cells growing in suspension by utilizing automated micro-bioreactors, platforms of multiple low-volume bioreactors (<15 mL) operating in parallel [21], or systems with low control levels such as spinners and shake flasks [22]. Well-controlled cultivation microsystems, as a rule, have high costs, and for the following procedures, it is difficult to scale the best operating parameters. As insect cells are robust in terms of pH and dissolved oxygen tension, the implementation of the QbD tool in Schott flasks offers a cheap and scalable system with which to optimize VLP production by B/IC. Thus, the experimental efforts of this work were mostly addressed to compare Schott flasks and bench bioreactors with respect to the production of recombinant baculovirus and VLP using the rabies virus as a model.

1. Rabies VLP production

Rabies VLP were generated by co-infecting two monocistronic baculoviruses, each carrying the genetic information that codes for the production of a rabies virus matrix protein or glycoprotein. To generate these recombinant baculoviruses, the inserts were cloned into donor plasmids, which were transformed into E. coli bacteria for the transposition of these inserts into the bacmid (shortened genetic material of the baculovirus). These recombinant bacmids, carrying either the matrix protein gene or the glycoprotein gene, were transfected into Sf9 cells for the generation of recombinant baculoviruses. The construction and further details regarding the generation of recombinant baculoviruses are described in the work of Bernardino et al. [15]. When coinfecting the same cell, the matrix protein is expected to sprout (an intrinsic characteristic of this protein is to sprout spontaneously), taking with it the glycoprotein that was previously anchored to the cell membrane, thus generating the enveloped virus-like particle.

2. Growth kinetics and viability of uninfected Sf9 cells

**Figure 1:**
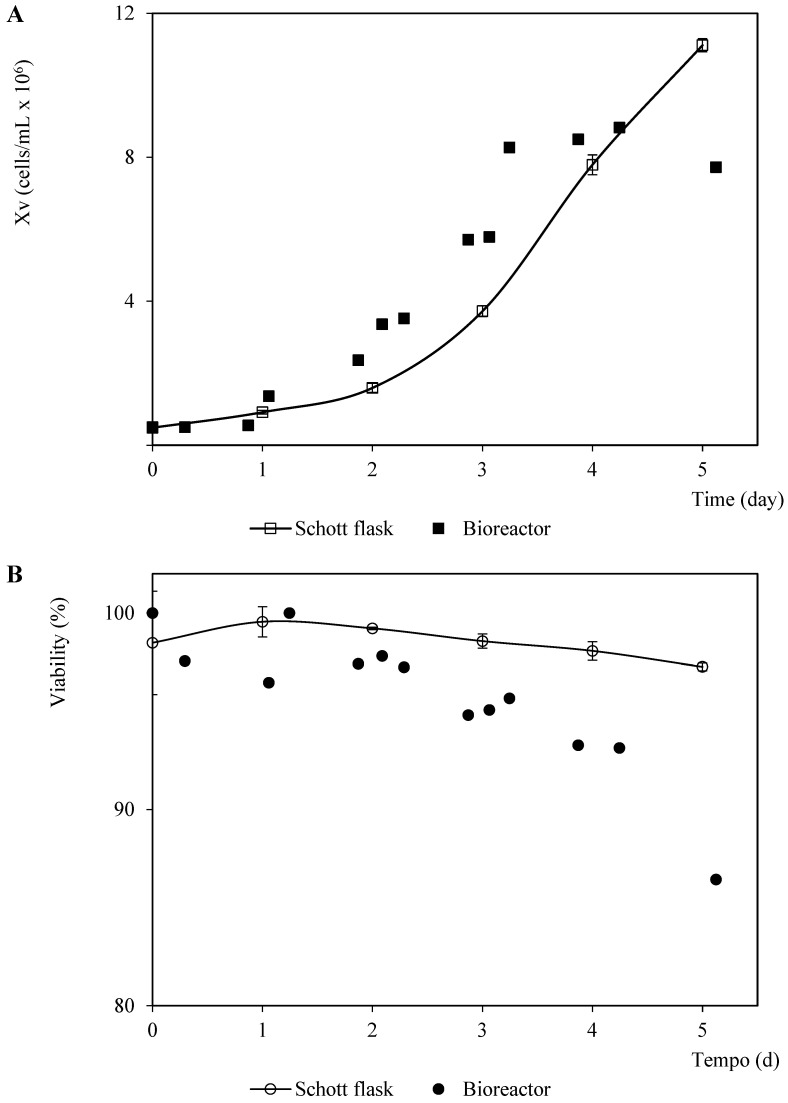
Patterns of viable cell density and viability corresponding to Sf9 cells in SF900 III culture medium in Schott flask and bioreactor. (**A**) Viable cell density pattern. (**B**) Viability pattern. The points and error bars from the Schott flask assays represent the average and the standard deviation of three repetitions, respectively.

Initially, the non-infected Sf9 growth kinetics in the SF900 III culture medium was monitored in Schott flasks and a bioreactor (Figure 1). A one-day lag phase was observed in the bioreactor assay, while cell growth in the Schott flask started in the exponential growth phase soon after inoculation, remaining in this phase until the fourth day of cultivation, with a maximum specific growth rate ($\mu_{m}$) of 0.696 day^−1^ (doubling time, $t_{d}$ equal to 23.9 h). These kinetic parameters were similar to those calculated from the exponential data (1–3 days) of the culture performed in the bioreactor ($\mu_{m}=0.703{\;\mathrm{day}}^{-1},{\;t}_{d}=23.7\;h$). The maximum cell concentration in the Schott flask was higher than that confirmed in the bioreactor (26% higher). In addition, the stationary phase in the culture carried out in the bioreactor was reached on the third day of the culture, while the assay in the Schott flask on the fourth culture day was still in the exponential growth phase (Figure 1). The peak cell density (PCD) reported for this cell line in SF900 III is within the 10–14 $\times 10^{6}$cells/mL range in the batch suspension culture [23]. The experiments performed in the same bioreactor, culture medium, cell line, and similar operation parameters showed a PCD equal to 10 $\times 10^{6}$cells/mL, which is close to the value observed for the Schott flask assay in this study [24]. Thus, the differences confirmed herein for this growth parameter in both culture systems could be associated with experimental errors (unstable dissolved oxygen control at the beginning of the assay, data not shown). In addition, the cell viability was higher than 85% throughout the assays, but the differences were more significant at the end of the culture times 1 × 10^6^.

3. Growth kinetics and cell death of Sf9 cells infected with BVM or BVG and coinfected with BVM and BVG

**Figure 2:**
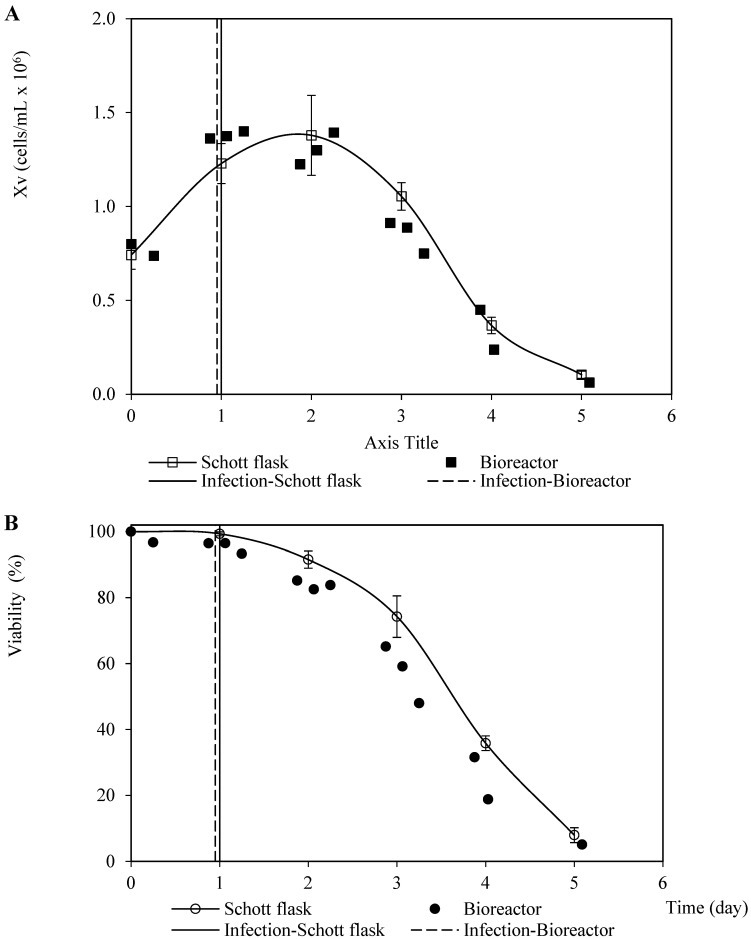
Patterns of viable cell density and viability corresponding to Sf9 cells infected with recombinant baculovirus carrying the rabies G glycoprotein at 0.1 pfu/cell MOI in Schott flask and bioreactor. (**A**) Viable cell density pattern. (**B**) Viability pattern. The points and error bars from Schott flask assays represent the average and the standard deviation of three repetitions, respectively.

**Figure 3:**
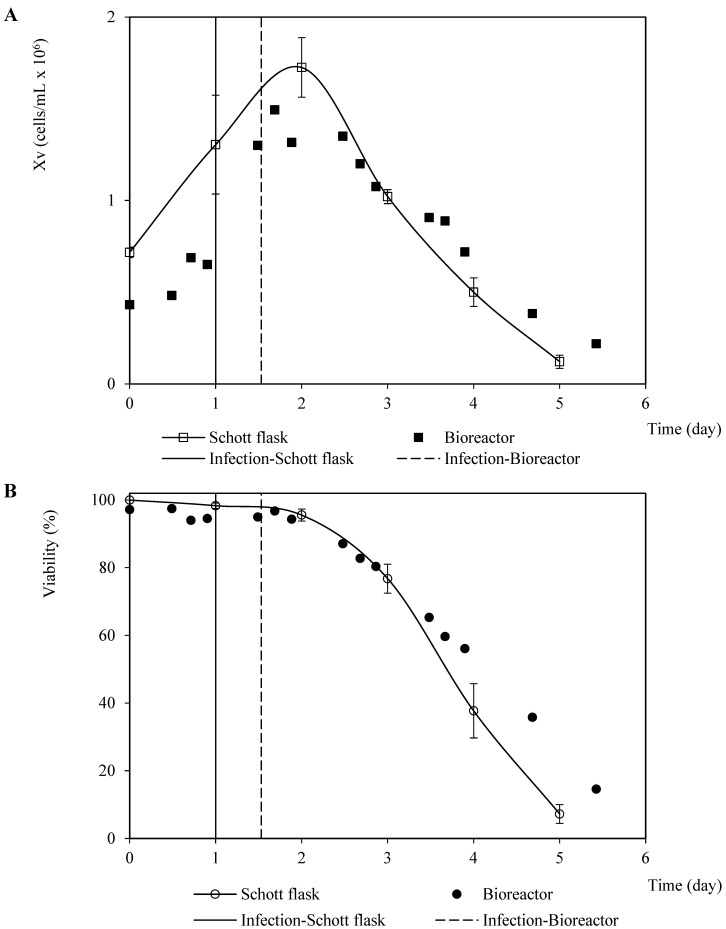
Patterns of viable cell density and viability corresponding to Sf9 cells infected with recombinant baculovirus carrying the rabies M protein at 0.1 pfu/cell MOI in Schott flask and bioreactor. (**A**) Viable cell density pattern. (**B**) Viability pattern. The points and error bars from Schott flask assays represent the average and the standard deviation of three repetitions, respectively.

Analogous patterns were observed concerning the viable cell density and viability associated with the infection assays for propagating the recombinant baculovirus carrying the rabies glycoprotein on Sf9 cells performed in the Schott flask and bioreactor (Figure 2). This finding suggests a minimal dependence of the baculovirus infection process on the dissolved oxygen tension and hydrodynamic configurations used in both culture systems. The cell death phase after infection confirmed a typical linear pattern with a similar instant cell death rate of 0.6 $\times 10^{6}$ cells/mL-day and 0.5 $\times 10^{6}$ cells/mL-day (average of three repetitions) for the bioreactor and Schott flask, respectively. This was caused by the changes in the specific cell death rate over time as the number of virions infecting the cells increases [25]. On the other hand, the referred patterns for the propagation derived from the recombinant baculovirus carrying rabies M protein were not as similar as those observed for the rabies glycoprotein, which occurred mostly during the growth phase before infection. This performance seems to have been caused by the difference in the inoculum cell density and TOI (Figure 3). However, the instant cell death rates after infection were close: 0.4 $\times 10^{6}$ cells/mL-day and 0.5$\times 10^{6}$ cells/mL-day (average of three repetitions) for the bioreactor and Schott flask, respectively.

The BVG and BVM coinfection assays performed to optimize the rabies VLP revealed similar profiles for viable cell density and viability after virus infection according to the BVM MOI. Cell death began immediately after infection at BVM MOI values of 1 and 3, regardless of the BVG/BVM ratio, but the cell death rate BVM MOI of 1 was higher than that relative to the BVM MOI of 3. Nevertheless, the assays with a BVM MOI of 5 showed a short growth phase (24 h) after infection; thereafter, the cell death rates were higher than those observed for BVM MOIs equal to 1 (Supplementary Materials, Figures S1–S3).

4. Propagation of recombinant baculoviruses

**Figure 4:**
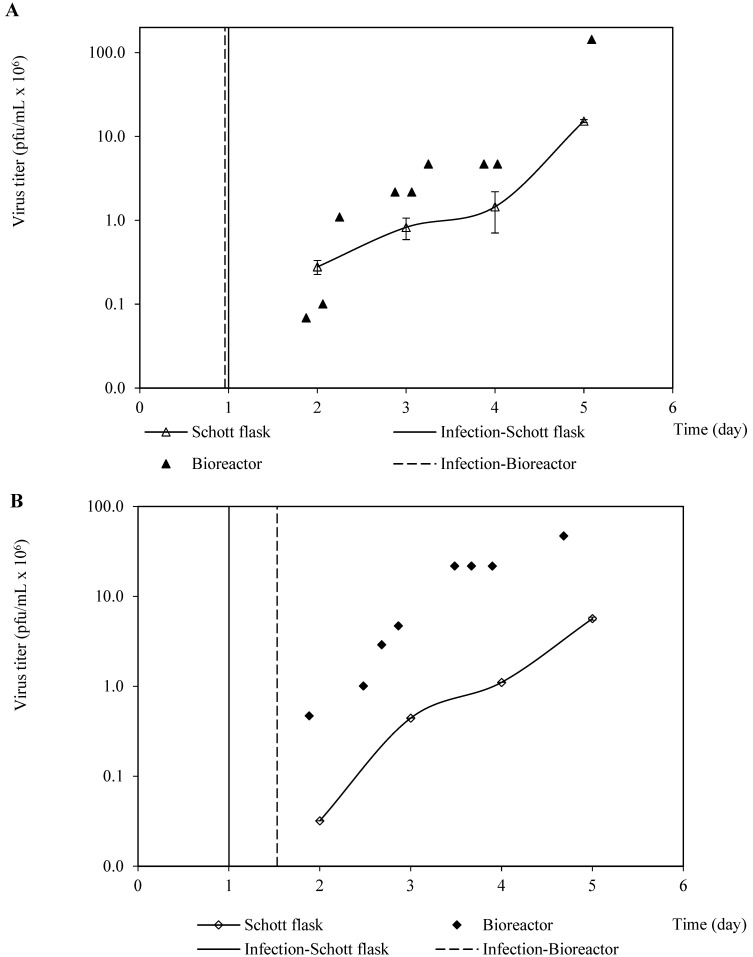
Kinetics of recombinant baculovirus production (pfu/cell) in Schott flask and bioreactor. (**A**) Kinetic production patterns of recombinant baculovirus carrying the rabies G glycoprotein. (**B**) Kinetic production patterns of recombinant baculovirus carrying the rabies M protein.

In addition, the production kinetics profiles of both rabies-recombinant baculoviruses were similar between the Schott flask and bioreactor culture systems in terms of shape, but the virus titers were higher in the bioreactor cultures than those confirmed in the Schott flasks (Figure 4). This finding, which is not in harmony with the confirmed similarity of the profiles of viable cell density and cell viability, could have been caused by the discrepancies in the hydrodynamic features and the chemical environments from both culture systems. These differences would negatively impact the virus’s stability, causing inactivation in the Schott flask system. However, as the virus profile propagations are qualitatively similar, the Schott flasks could be used as a suitable culture system platform for the screening and optimization of critical parameters related to baculovirus propagation, and likely for VLP production as well. Recently, a comparative study among culture systems concerning the creation of VLP demonstrated qualitatively similar VLP generation profiles in Shake flasks and a benchtop bioreactor [13]. Then, the Schott flasks were used to optimize the BVM MOI and BVG/BVM MOIs ratio, using the harvest time to maximize the VLP yield.

5. Quantification of rabies matrix protein by Dot-blot

The primary data from the rabies M protein production kinetics monitored by Dot-blot allowed us to identify sigmoidal expression curves. This curve shape has been reported for other heterologous proteins [26]. Following a general rule, the protein concentration plateau was confirmed on the fourth day after coinfection. The maximum values of the rabies M protein concentration were around 600 ng/mL and were achieved through different combinations of HT and BVM MOI values in all the assessed BVG/BVM MOI ratios. The maximum concentration of M protein was reached 96 h after infection using a BVG/BVM MOI ratio of 1.5 and an M MOI of 1. When BVG/BVM MOI ratios of 2 and 2.5 were used, the peak concentration of M protein was at MOIs of 1 and 3, respectively (Supplementary Materials, Figures S4–S6). The statistical modeling of the rabies M protein concentration (RVM) as a function of the BVM MOI and BVG/BVM ratio and HT demonstrated a marked positive influence of HT, while an appositive effect was confirmed for BVM MOI, as both factors increased. Moreover, the BVG/BVM MOI ratio within the considered range (BVG/BVM between 1.5 and 2.5) had no significant impact on the RVM. The negative effect of the HT quadratic term describes the plateau identified from the primary data (Table 3). The influence of each parameter on the RVM might also be confirmed more clearly by the perturbation plot (Supplementary Materials, Figure S7)

6. Quantification of rabies glycoprotein by Dot-blot and ELISA

The RVGP concentration (determined by Dot-blot) model as a function of the assessed critical parameters again demonstrated the low impact of the BVG/BVM MOI ratio and a negative effect of BVM MOI at high values. Conversely, the HT had a positive effect at middle and high values within the considered range (harvest time between 24 and 96 h). The maximum concentration of RVGP was reached 120 h after infection using a BVG/BVM MOI ratio of 2.5 and a BVM MOI of 3. When BVG/BVM MOI ratios of 1.5 and 2 were used, the peak concentration of RVGP was at BVM MOIs of 1 and 3, respectively (Table 3, Supplementary Materials, Figures S8–S11). When the RVGP concentration was determined by ELISA, a repetitive pattern was observed in most of the cases, in which a plateau was achieved after 72 h from the moment of infection. The observed maximum concentration values of this protein determined by ELISA were obtained at combinations of the BVG/BVM MOI ratio and BVM MOI of 1.5, 3 (1303 ng/mL) and 2.0, and 3 (1240 ng/mL) (Supplementary Materials, Figures S12–S14). According to the statistical model regarding RVGP concentration by ELISA and its associated factor perturbation graph, the most significant factor for RVGP concentration was HT, whereas the BVM MOI and the BVG/BVM MOI ratio had a slight positive and negative influence, respectively, within the assessed parameter range (Table 3, Supplementary Materials, Figure S15).

7. Optimization of the production of rabies virus-like particles

**Figure 5:**
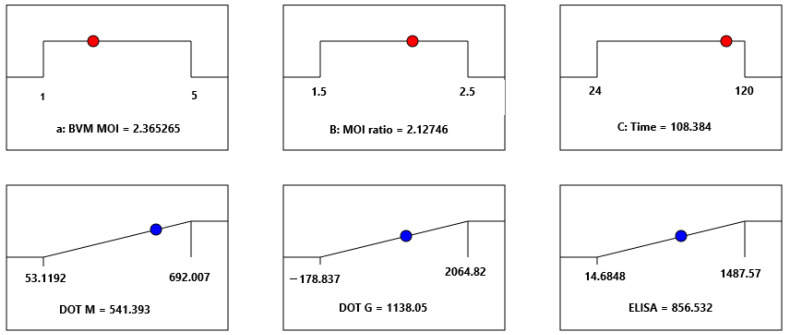
Ramp plots depicting the optimal solution that best satisfies the conditions described in Table 2. This solution was determined by the desirability function implemented in Design Expert^®^ version 13 software.

Once the protein concentrations were statistically modeled, a multiple optimization procedure was performed following the criteria depicted in Table 2. The best combination of BVM MOI, the BVG/BVM MOI ratio, and HT was 2.36 pfu/cell, 1.5 MOI ratio, and 108 h, respectively (Figure 5). In the latter figure, it is possible to see the position of the optimal condition in each parameter within the studied ranges of the BVG/BVM MOI ratio, BVM MOI, and harvest time (red balls). By observing the blue spheres, it is possible to see the numerical value of each parameter in the optimal condition. Since the effective impact of the BVG/BVM MOI ratio was not significant within the 1.5–2.0 range (Supplementary Materials, Figures S7, S11, and S15), and due to the level of accuracy of the virus titration method and the high cost of recombinant baculovirus production, the combination assessed in the benchtop stirred-tank bioreactor yielded a BVM MOI of 2.0 pfu/cell and a BVG/BVM MOI ratio of 1.5, while the degree of VLP production was monitored around 4 days after coinfection. This pair of critical parameters was similar to that previously reported for Chimeric Rabies VLP bearing the same proteins and membrane-anchored granulocyte-macrophage colony-stimulating factor but using a shake flask as a culture system [27].

8. Comparison of the optimal conditions with the implemented conditions

**Figure 6:**
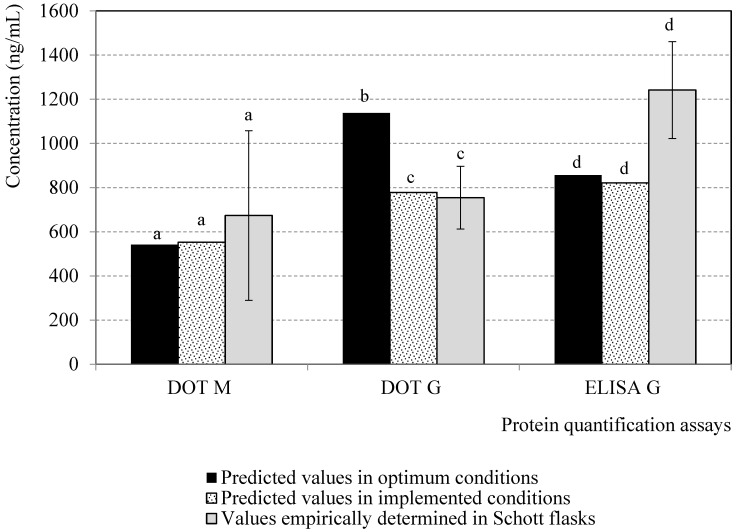
Predicted values of protein concentrations in Schott flasks for optimal conditions (Figure 5) and for those that were implemented in bench bioreactor (BVM MOI of 2.0 pfu/cell, 1.5 BVG/BVM MOI ratio), and corresponding empirical values in the last conditions using stirred-tank bioreactor as culture system. Columns represent predicted values by statistical models or the average from three experimental repetitions. Error bars depict the standard deviation from triplicate empirical runs. The letters represent statistically similar values for each protein concentration quantification technique.

To confirm the analogous performance with respect to protein expression, the fitted models were used to predict the M and G concentrations via DOT, as well as the G concentration via ELISA, for both sets of operating conditions (optimum and implemented ones). The M and G concentrations determined by DOT and ELISA, respectively, were similar, and the G concentration determined by DOT for the implemented conditions was 32 % lower than that predicted for the optimum conditions (Figure 6). The implemented parameters set in the bioreactor were also empirically assessed in the Schott flasks to assess the prediction capacities of the models. The predicted and experimental concentrations for M and G proteins via DOT and ELISA were statistically similar (Hypotheses test, α > 0.05) (Figure 6), which demonstrated the fitting of the models to the experimental data over the evaluated factors domain.

**Figure 7:**
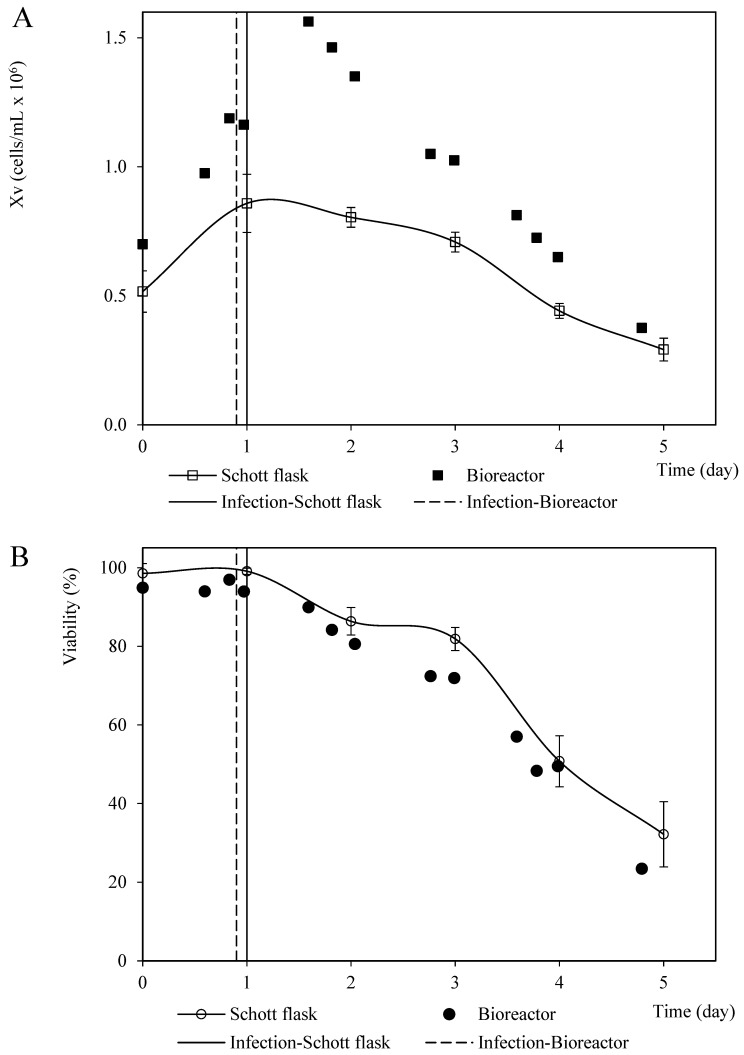
Patterns of viable cell density and viability corresponding to Sf9 cells coinfected with recombinant baculovirus carrying the rabies G glycoprotein and M protein MOI at 3 pfu/cell and 2 pfu/cell, respectively, in Schott flask and bioreactor. (**A**) Viable cell density pattern. (**B**) Viability pattern. The points and error bars from Schott flask assays represent the average and the standard deviation of three repetitions, respectively.

The viable cell density profiles from the coinfection assays performed in the Schott flasks and bioreactor at a BVM MOI of 2.0 pfu/cell and a 1.5 BVG/BVM MOI ratio were similar in terms of shape but were quantitatively divergent. This phenomenon was likely caused by the differences between the initial cell density and inoculum quality. The instant cell death rates were 0.4 $\times 10^{6}$ cells/mL per day and 0.2 $\times 10^{6}$ cells/mL per day for the assays performed in the bioreactor and a Schott flask, respectively. However, the viability profiles were quantitatively homologous in both culture systems (Figure 7).

**Figure 8:**
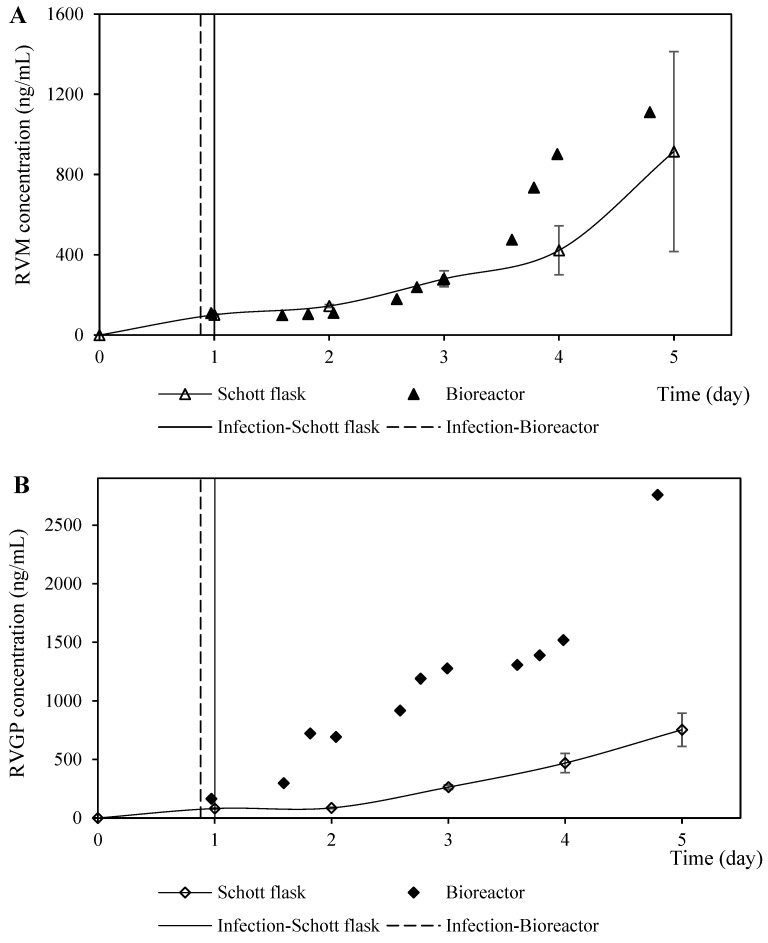

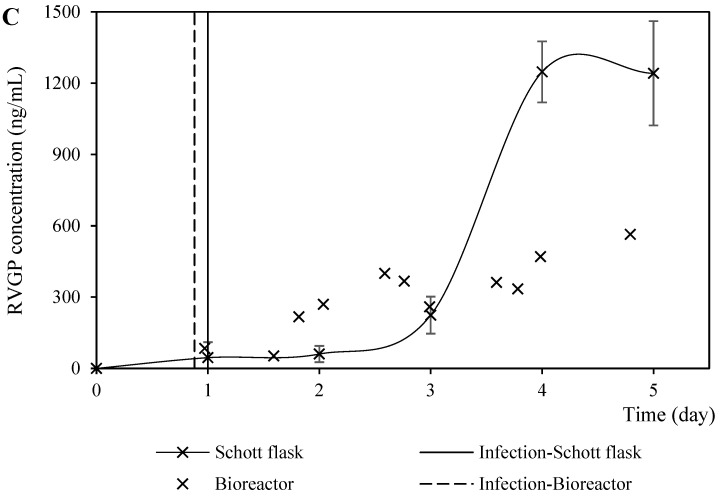
Rabies protein expression profiles from coinfection performed in Schott flasks and bioreactor at BVM MO of 2.0 pfu/cell and a BVG/BVM MOI ratio of 1.5. (**A**) Kinetics profiles of rabies matrix protein (RVM) production (measured by the signal intensity of the dot blot membrane labeled with anti-RVM antibody). (**B**) Kinetics profiles of rabies G glycoprotein (RVGP) production (measured by the signal intensity of the dot blot membrane labeled with anti-RVGP antibody). (**C**) Kinetics profiles of rabies G glycoprotein (RVGP) production (measured by ELISA).

The protein expression profiles in the coinfection assays in both the assessed culture systems were similar in terms of RVM but quite different for the RVGP determined by Dot blot (with an antibody that detects monomers) and ELISA (with an antibody that detects trimers) (Figure 8). The values of the protein concentration of RVM and RVGP determined by Dot-blot (BVM MOI of 2.0 pfu/cell and a 1.5 BVG /BVM MOI ratio) in the Schott flask were within the expected values, while the RVGP concentration determined by ELISA was lower than the projected values (Figure 5 and Figure 8). These discrepancies could be related to the loss of the trimeric assembly of RVGP—which was quantified by ELISA—in the bioreactor caused by its hydrodynamic properties or chemical background. The RVGP trimers are the main ones responsible for the induction of virus-neutralizing antibodies [27]. However, it has also been described that monomeric glycoprotein is also able to protect mice against a rabies virus challenge [28]. Therefore, additional experiments need to be accomplished using the bioreactor to configure the parameters associated with agitation and aeration for increasing the RVGP concentration determined by ELISA.

**Figure 9:**
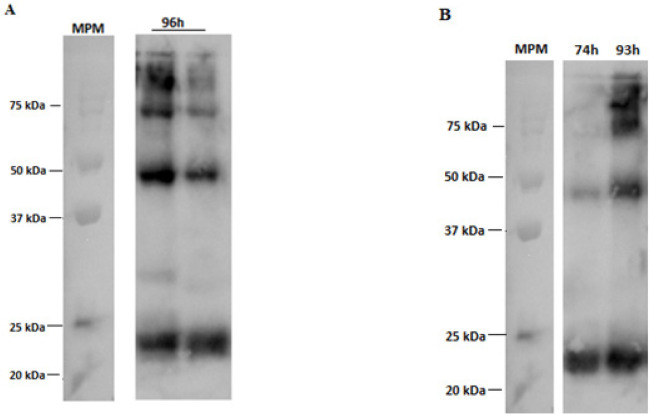
Western blotting labeled with anti-RVM antibody from co-infection assays (M MOI of 2 pfu/cell, G MOI of 3 pfu/cell). (**A**) 96 h post-infection Schott flask samples. (**B**) Samples from 74 and 93 h post-infection in the bioreactor. MPM = molecular weight marker.

The Western blotting of RVM, as part of an immunochemical characterization, revealed an intense protein spot around 25 kDa in both culture systems, which is yet another confirmation of the biological activity of the RVM expressed in the coinfection assays (Figure 9) [15]. It is also possible to analyze bands at the height of 50, 75, 80, and 120 kDa, which suggests that RVM may be anchoring RVGP to VLP forms.

**Figure 10:**
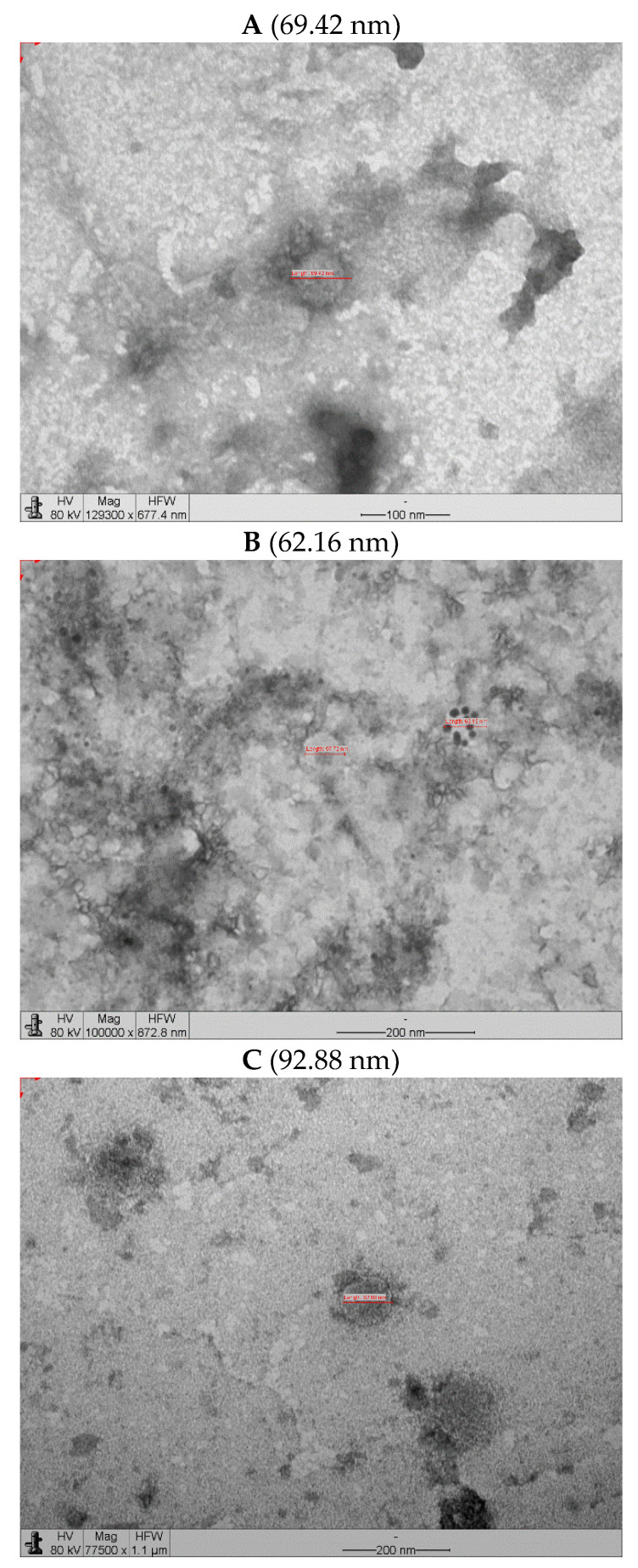

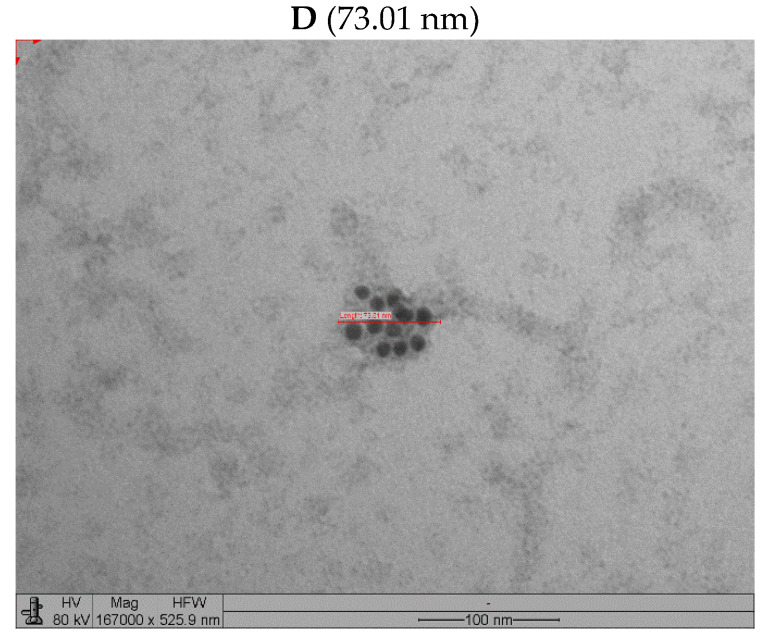
Transmission electron micrographs of VLP obtained in Schott flask and bioreactor at BVM MOI of 2 pfu/cell and BVG MOI of 3 pfu/cell. (**A**) Micrograph of VLP produced in Schott flask without immunostaining. (**B**) Micrograph of VLP produced in Schott flask with immunostaining (ant- G). (**C**) Micrograph of VLP produced in benchtop bioreactor without immunostaining. (**D**) Micrograph of VLP produced in benchtop bioreactor with immunostaining (anti-G).

The VLP produced in the Schott flasks were slightly lower than those produced in the bench-top bioreactor (Figure 10). Figure 10A,B show micrographs of the structures labeled with 10 nm colloidal gold for the anti-mouse antibody used for the samples incubated with the anti-glycoprotein antibody. The particle sizes were within the 62–93 nm range. The sizes were almost half those reported for equivalent rabies VLP (150–190 nm) obtained at low MOIs [15] and those produced at similar MOIs of RVM and RVGP but also incorporating another protein (180–200 nm) [27]. 

## 4. Conclusions

The current study demonstrated that the utilization of Schott flasks and a benchtop bioreactor at the conditions used herein are equivalent to the infection process of recombinant baculovirus in Sf9 cells. According to the results, the hydrodynamic and chemical differences in both culture systems seem to mostly impact the virus and virus-like particles’ stability as well as their integrity after they are produced. Future works aiming to adjust the operation’s parameters in both systems need to be carried out to obtain similar performance and improve the Schott flask’s scalability. However, the Schott flask is a suitable culture system for the screening and optimization of critical parameters involved in monocistronic baculovirus infection and coinfections used to yield rabies VLP. Further studies assessing the immunogenicity of the particles will need to be performed so that our produced VLP can be used as a vaccine candidate. Our research group intends to perform these studies through in vitro and in vivo tests to analyze the immunogenic and antigenic capacity of the VLP to ensure that we are producing rabies virus-like particles capable of generating a protective response.

## Figures and Tables

**Table 1:** The ratio between the MOIs as well as MOI values for BVs used in the co-infection assays.

Co-Infection Experiment Blocks According to Ratio BVM/BVG					
1		2		3	
Ratio BVG/BVM					
1.5		2.0		2.5	
BV MOIs					
BVM	BVG	BVM	BVG	BVM	BVG
1.0	1.5	1.0	2.0	1.0	2.5
3.0	4.5	3.0	6.0	3.0	7.5
5.0	7.5	5.0	10.0	5.0	12.5

**Table 2:** Criteria implemented in Derringer and Suich’s desirability function (Design Expert^®^ version 13 software) for rabies VLP production optimization in co-infections blocks 1, 2, and 3 (Table 1).

Response Variable	Goal	Importance *
M Protein concentration by Dot blot	Maximize	+++
G Protein concentration by Dot blot	Maximize	++++
G Protein concentration by ELISA	Maximize	+++++

Importance * = specifies the relative relevance of one goal versus others. G protein concentration by ELISA was assigned the highest importance because this assay quantifies the trimeric immunogenic form of G protein.

**Table 3:** Summary of adjusted statistical quadratic models in coded factors (range of −1 to +1) for protein concentrations by dot blot and ELISA derived from overall experimental design including all co-infection experiments, namely, 1, 2, and 3 (Table 1). The critical parameters assessed were BVM MOI (A), BVG/BVM MOI ratio (B), and HT (C). The adjusted quadratic models and terms included in them were statistically significant (*p* < 0.05).

Protein Concentration Quantification	Mathematical Equations Using Coded Factors
RVM (ng/mL) (Dot blot)	$M\;\mathrm{concentration}=437.39-64.42A-6.75B+191.59C-26.76\mathrm{BC}-44.32A^{2}-87.65C^{2}$
RVGP (ng/mL) (Dot blot)	$G\;\mathrm{concentration}=1139.74-241.62A+155.01B+260.06C+115.78\mathrm{BC}-330.94A^{2}-189.98B2-507.76C^{2}$
RVGP (ng/mL) (ELISA)	G concentration=652.02+69.93A−36.15B+426.98C−68.44AB+45.27AC−43.05 BC+ 52.86$A^{2}$—138.05 C^2^

## Data Availability

Not applicable.

## Supplementary Materials

The following are available online at https://www.mdpi.com/article/10.3390/vaccines11010039/s1, Figure S1: Kinetics profiles of viable cell density and viability from Sf9 cells co-infected with recombinant baculoviruses carrying the rabies glycoprotein (BVG) and matrix protein (BVM) with a ratio between BVG and BVM of 1.5 using Schott flask as culture system. Figure S2: Kinetics profiles of viable cell density and viability from Sf9 cells co-infected with recombinant baculoviruses carrying the rabies glycoprotein (BVG) and matrix protein (BVM) with a ratio between BVG and BVM of 2.0 using Schott flask as culture system. Figure S3: Kinetics profiles of viable cell density and viability from Sf9 cells co-infected with recombinant baculoviruses carrying the rabies glycoprotein (BVG) and matrix protein (BVM) with a ratio between BVG and BVM of 2.5 using Schott flask as culture system. Figure S4: Kinetics profiles of rabies matrix protein (RVM) production (measured by the signal intensity of the dot blot membrane labeled with anti-RVM antibody) from Sf9 cells co-infected with recombinant baculoviruses carrying the rabies glycoprotein (BVG) and matrix protein (BVM) with a ratio between BVG and BVM of 1.5 using Schott flask as culture system. Figure S5: Kinetics profiles of rabies matrix protein (RVM) production (measured by the signal intensity of the dot blot membrane labeled with anti-RVM antibody) from Sf9 cells co-infected with recombinant baculoviruses carrying the rabies glycoprotein (BVG) and matrix protein (BVM) with a ratio between BVG and BVM of 2.0 using Schott flask as culture system. Figure S6: Kinetics profiles of rabies matrix protein (RVM) production (measured by the signal intensity of the dot blot membrane labeled with anti-RVM antibody) from Sf9 cells co-infected with recombinant baculoviruses carrying the rabies glycoprotein (BVG) and matrix protein (BVM) with a ratio between BVG and BVM of 2.5 using Schott flask as culture system. Figure S7: Perturbation graph of BVM MOI (A), BVG/BVM MOI ratio (B), and HT (C) on rabies M protein concentration (RVM) (determined by Dot-blot) modelled statistically. Figure S8: Kinetics profiles of rabies G glycoprotein (RVGP) production (measured by the signal intensity of the dot blot membrane labeled with anti-RVGP antibody) from Sf9 cells co-infected with recombinant baculoviruses carrying the rabies glycoprotein (BVG) and matrix protein (BVM) with a ratio between BVG and BVM of 1.5 using Schott flask as culture system. Figure S9: Kinetics profiles of rabies G glycoprotein (RVGP) production (measured by the signal intensity of the dot blot membrane labeled with anti-RVGP antibody) from Sf9 cells co-infected with recombinant baculoviruses carrying the rabies glycoprotein (BVG) and matrix protein (BVM) with a ratio between BVG and BVM of 2.0 using Schott flask as culture system. Figure S10: Kinetics profiles of rabies G glycoprotein (RVGP) production (measured by the signal intensity of the dot blot membrane labeled with anti-RVGP antibody) from Sf9 cells co-infected with recombinant baculoviruses carrying the rabies glycoprotein (BVG) and matrix protein (BVM) with a ratio between BVG and BVM of 2.5 using Schott flask as culture system. Figure S11: Perturbation graph of BVM MOI (A), BVG/BVM MOI ratio (B), and HT (C) on rabies G glycoprotein concentration (RVGP) (determined by Dot-blot) modelled statistically. Figure S12: Kinetics profiles of rabies G glycoprotein (RVGP) production (measured by ELISA) from Sf9 cells co-infected with recombinant baculoviruses carrying the rabies glycoprotein (BVG) and matrix protein (BVM) with a ratio between BVG and BVM of 1.5 using Schott flask as culture system. Figure S13: Kinetics profiles of rabies G glycoprotein (RVGP) production (measured by ELISA) from Sf9 cells co-infected with recombinant baculoviruses carrying the rabies glycoprotein (BVG) and matrix protein (BVM) with a ratio between BVG and BVM of 2.0 using Schott flask as culture system. Figure S14: Kinetics profiles of rabies G glycoprotein (RVGP) production (measured by ELISA) from Sf9 cells co-infected with recombinant baculoviruses carrying the rabies glycoprotein (BVG) and matrix protein (BVM) with a ratio between BVG and BVM of 2.5 using Schott flask as culture system. Figure S15: Perturbation graph of BVM MOI (A), BVG/BVM MOI ratio (B), and HT (C) on rabies G glycoprotein concentration (RVGP) (determined by ELISA) modelled statistically.
