# Supplementary material for: Performance Comparison of Recombinant Baculovirus and Rabies Virus-like Particles production Using Two Culture Platforms
Source: Vaccines (Basel). 2022 Dec 24;11(1):39. doi: 10.3390/vaccines11010039 (PMC9867115; doi:10.3390/vaccines11010039)

Supplementary material

Figure S1. Kinetics profiles of viable cell density and viability from Sf9 cells co-infected with recombinant baculoviruses carrying the rabies glycoprotein (BVG) and matrix protein (BVM) with a ratio between BVG and BVM of 1.5 using Schott flask as culture system. A) Coinfection 1: BVM 1 pfu/cell – BVG 1.5 pfu/cell. B) Coinfection 2: BVM 3 pfu/cell – BVG 4.5 pfu/cell. C) Coinfection 3: BVM 5 pfu/cell – BVG 7.5 pfu/cell. The points depict the average from three repetitions. Error bars represent the standard deviation from repetitions.

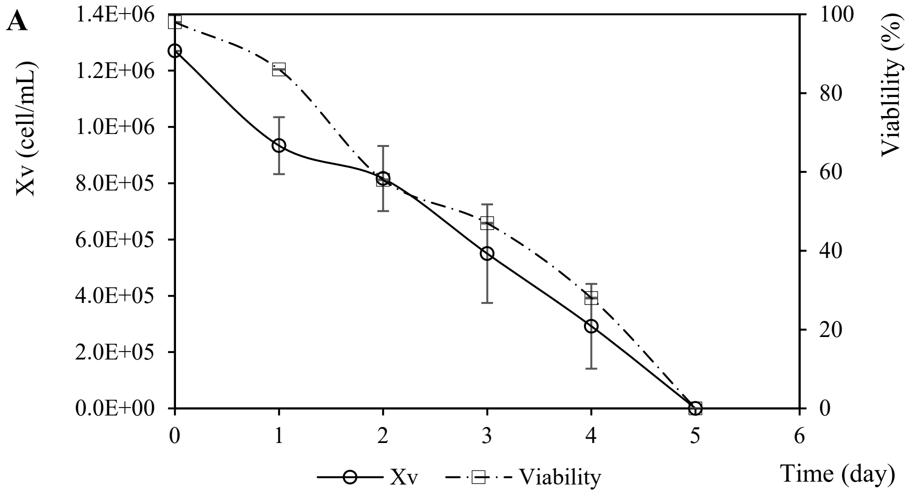

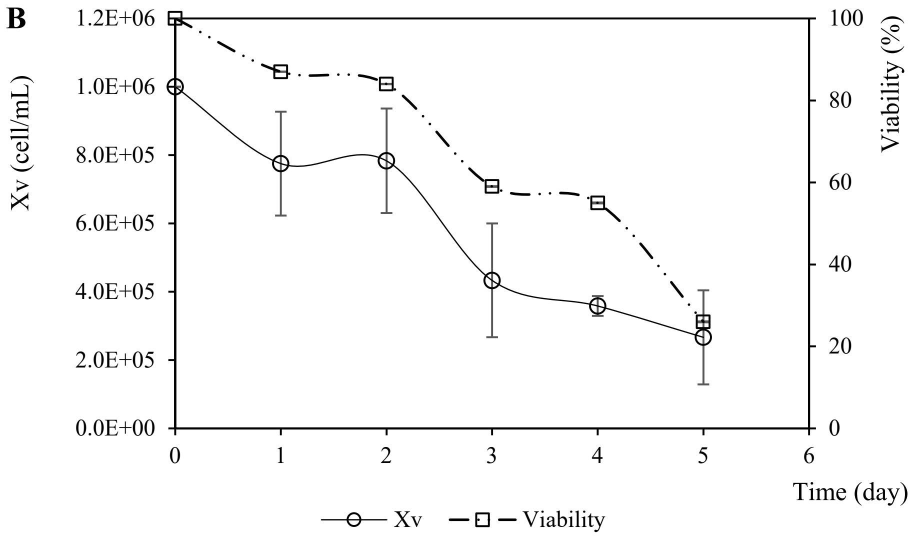

C

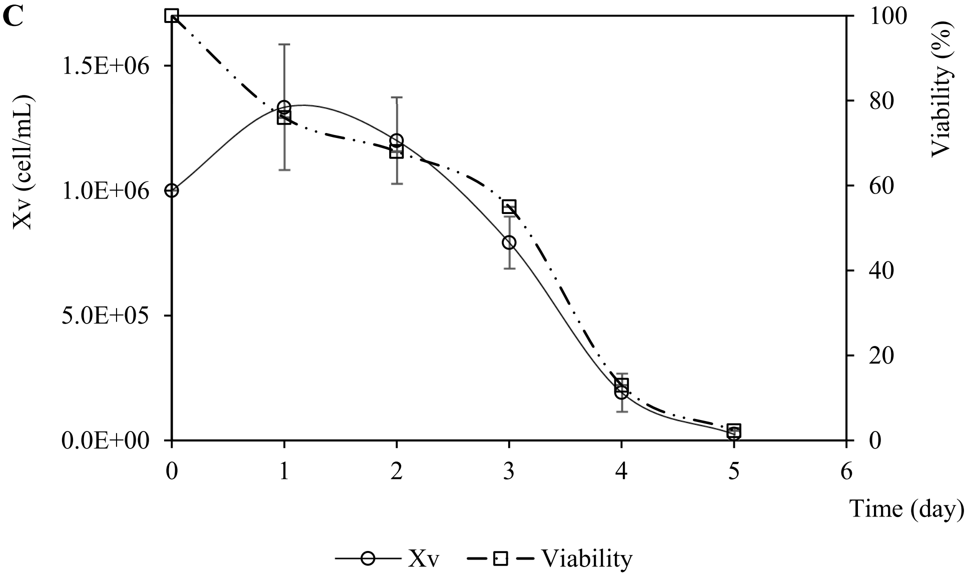
1.5E+06
1.0E+06
5.0E+05
0.0E+00
Xv (cell/mL)
100
80
60
40
20
0
Viability (%)
0
1
2
3
4
5
6
Time (day)
Xv
Viability

Figure S2. Kinetics profiles of viable cell density and viability from Sf9 cells co-infected with recombinant baculoviruses carrying the rabies glycoprotein (BVG) and matrix protein (BVM) with a ratio between BVG and BVM of 2.0 using Schott flask as culture system. A) Coinfection 1: BVM 1 pfu/cell – BVG 2.0 pfu/cell. B) Coinfection 2: BVM 3 pfu/cell – BVG 6.0 pfu/cell. C) Coinfection 3: BVM 5 pfu/cell – BVG 10.0 pfu/cell. The points depict the average from three repetitions. Error bars represent the standard deviation from repetitions.

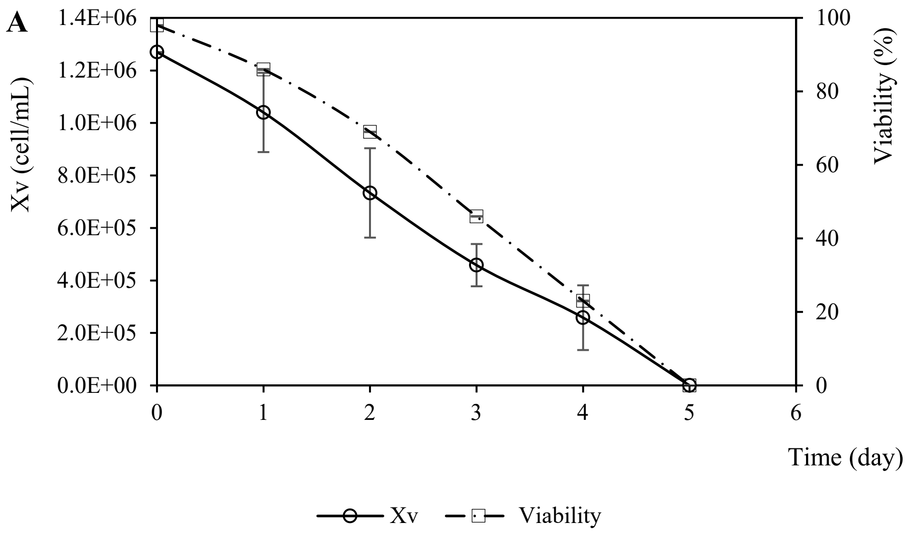

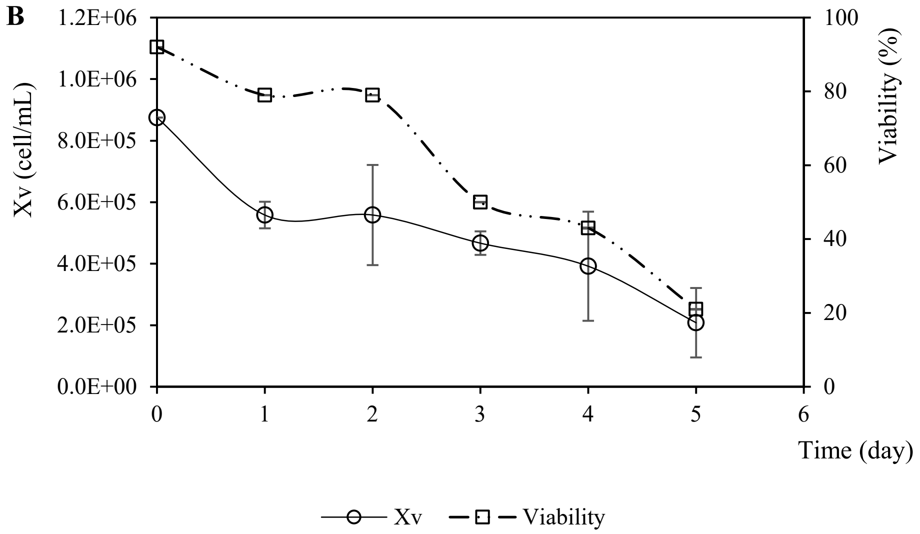

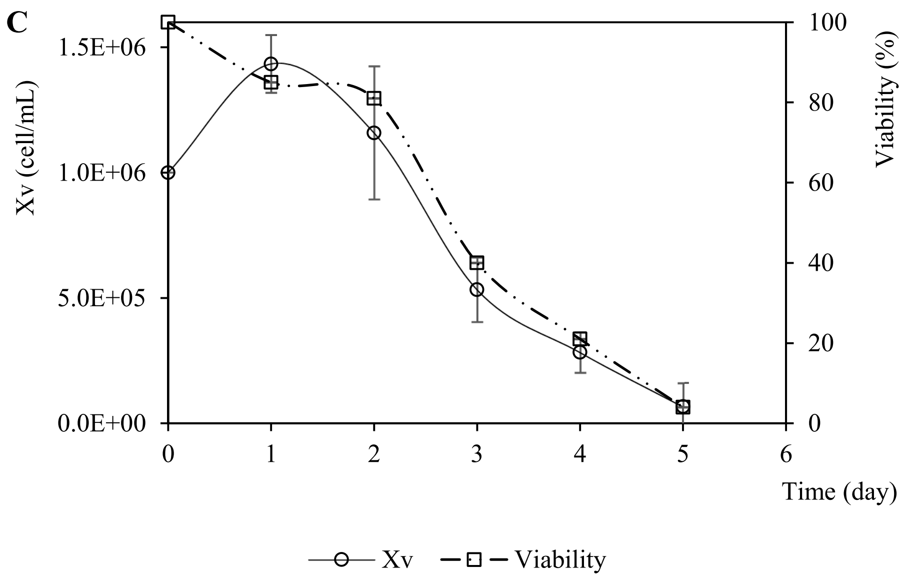

C
Xv (cell/mL)
1.5E+06
1.0E+06
5.0E+05
0.0E+00
Viability (%)
100
80
60
40
20
0
0
1
2
3
4
5
6
Time (day)
Xv
Viability

Figure S3. Kinetics profiles of viable cell density and viability from Sf9 cells co-infected with recombinant baculoviruses carrying the rabies glycoprotein (BVG) and matrix protein (BVM) with a ratio between BVG and BVM of 2.5 using Schott flask as culture system. A) Coinfection 1: BVM 1 pfu/cell – BVG 2.5 pfu/cell. B) Coinfection 2: BVM 3 pfu/cell – BVG 7.5 pfu/cell. C) Coinfection 3: BVM 5 pfu/cell – BVG 12.5 pfu/cell. The points depict the average from three repetitions. Error bars represent the standard deviation from repetitions.

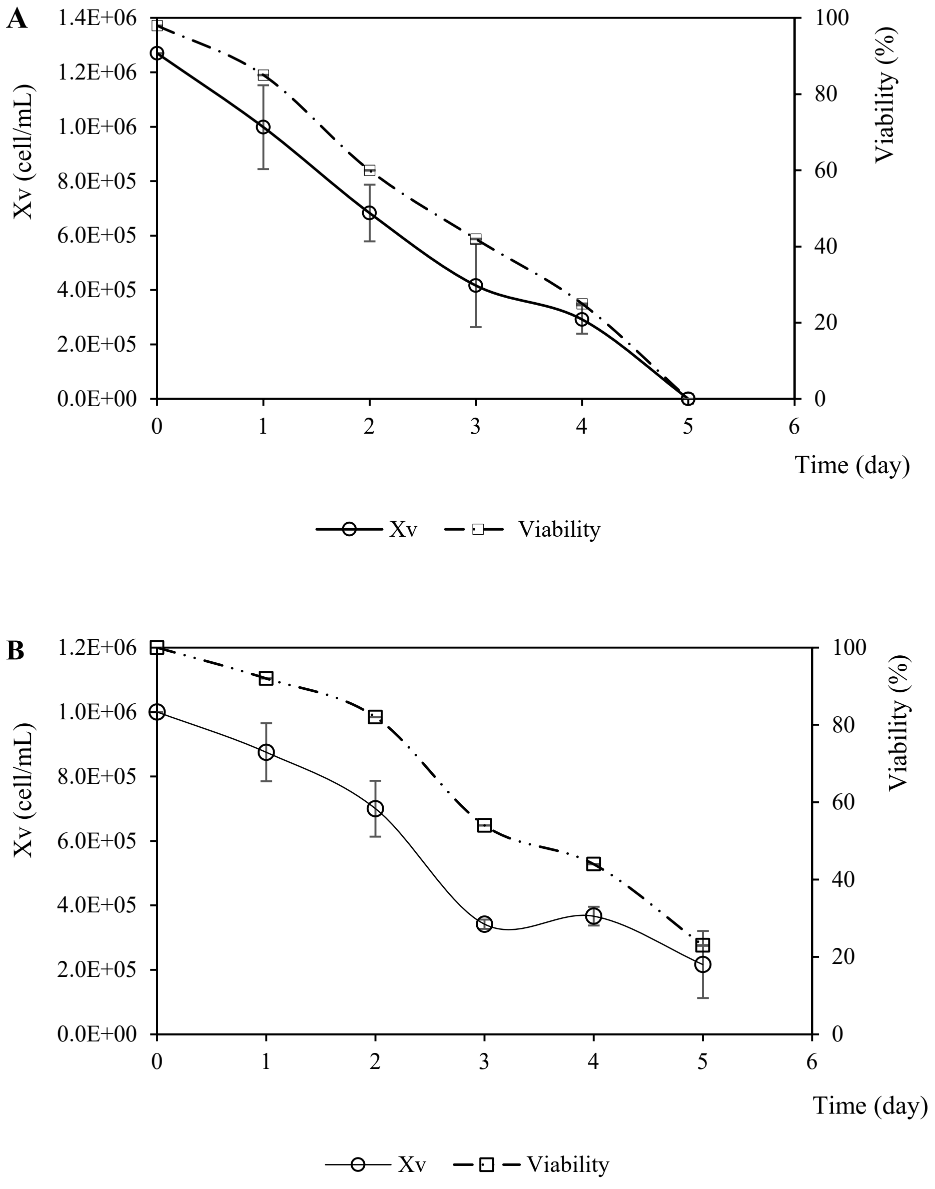

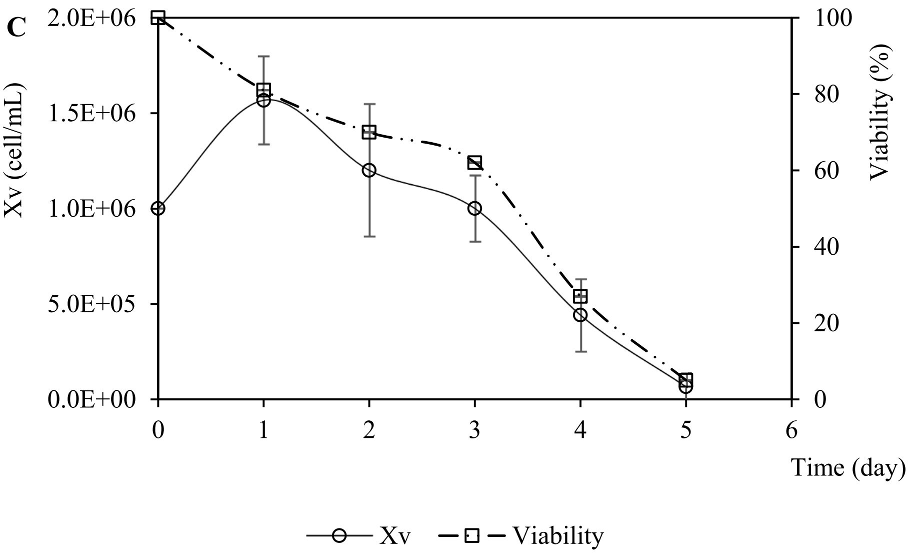

C
2.0E+06
1.5E+06
1.0E+06
5.0E+05
0.0E+00
Xv (cell/mL)
100
80
60
40
20
0
Viability (%)
0
1
2
3
4
5
6
Time (day)
Xv
Viability

Figure S4. Kinetics profiles of rabies matrix protein (RVM) production (measured by the signal intensity of the dot blot membrane labeled with anti-RVM antibody) from Sf9 cells co-infected with recombinant baculoviruses carrying the rabies glycoprotein (BVG) and matrix protein (BVM) with a ratio between BVG and BVM of 1.5 using Schott flask as culture system. A) Coinfection 1: BVM 1 pfu/cell – BVG 1.5 pfu/cell. B) Coinfection 2: BVM 3 pfu/cell – BVG 4.5 pfu/cell. C) Coinfection 3: BVM 5 pfu/cell – BVG 7.5 pfu/cell. The points depict the average from three repetitions. Error bars represent the standard deviation from repetitions.

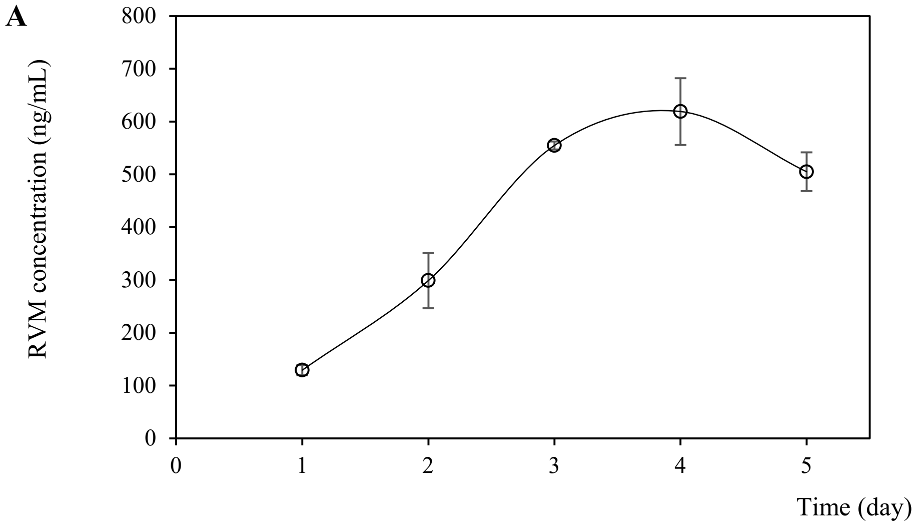

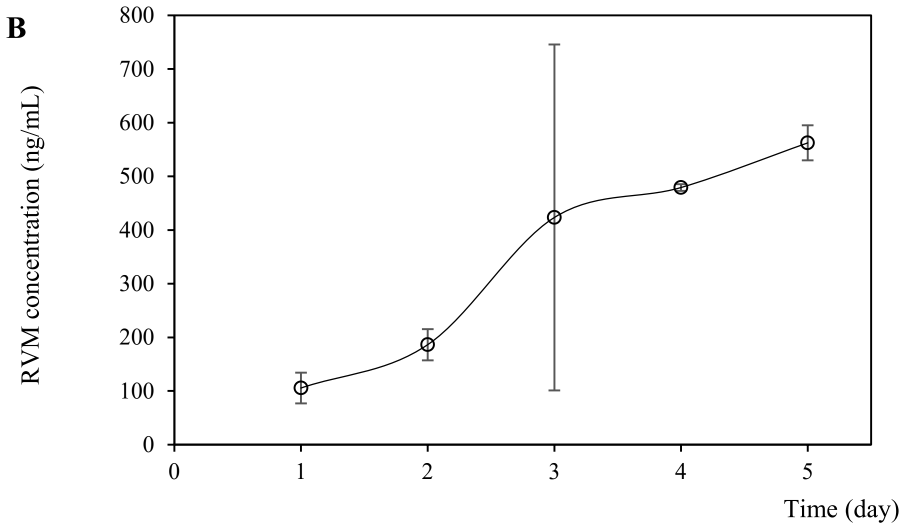

C

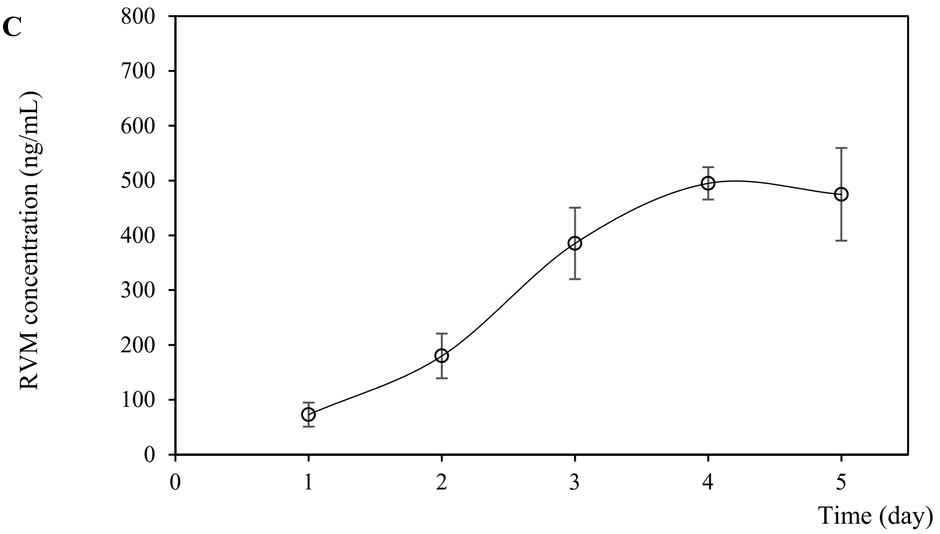
800
700
600
500
400
300
200
100
0
RVM concentration (ng/mL)
0
1
2
3
4
5
Time (day)

Figure S5. Kinetics profiles of rabies matrix protein (RVM) production (measured by the signal intensity of the dot blot membrane labeled with anti-RVM antibody) from Sf9 cells co-infected with recombinant baculoviruses carrying the rabies glycoprotein (BVG) and matrix protein (BVM) with a ratio between BVG and BVM of 2.0 using Schott flask as culture system. A) Coinfection 1: BVM 1 pfu/cell – BVG 2.0 pfu/cell. B) Coinfection 2: BVM 3 pfu/cell – BVG 6.0 pfu/cell. C) Coinfection 3: BVM 5 pfu/cell – BVG 10.0 pfu/cell. The points depict the average from three repetitions. Error bars represent the standard deviation from repetitions.

A

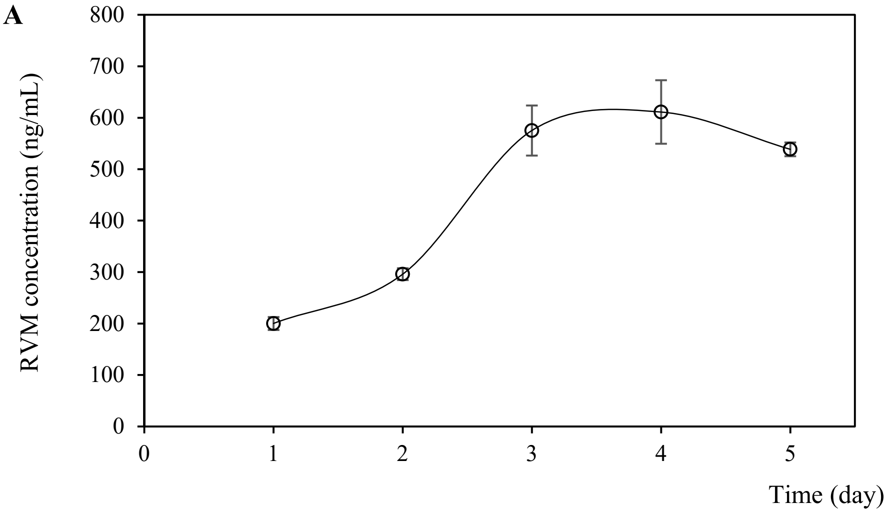

B

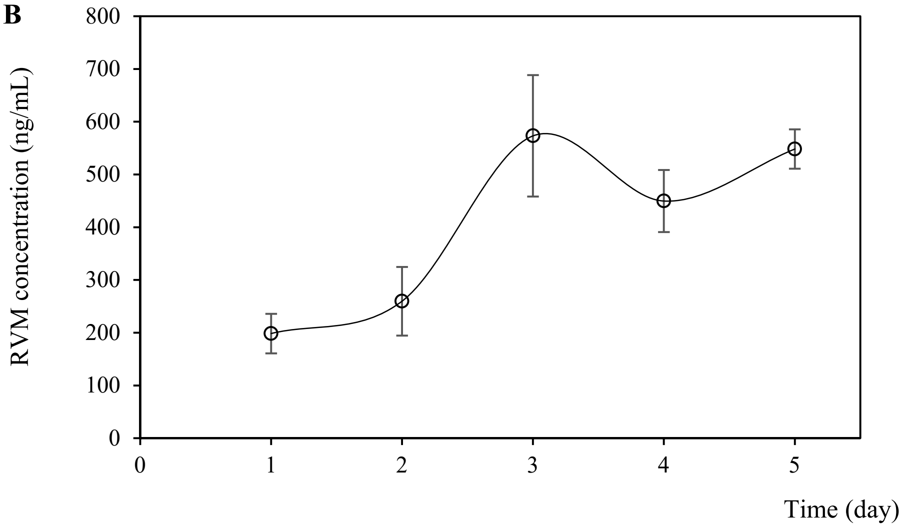

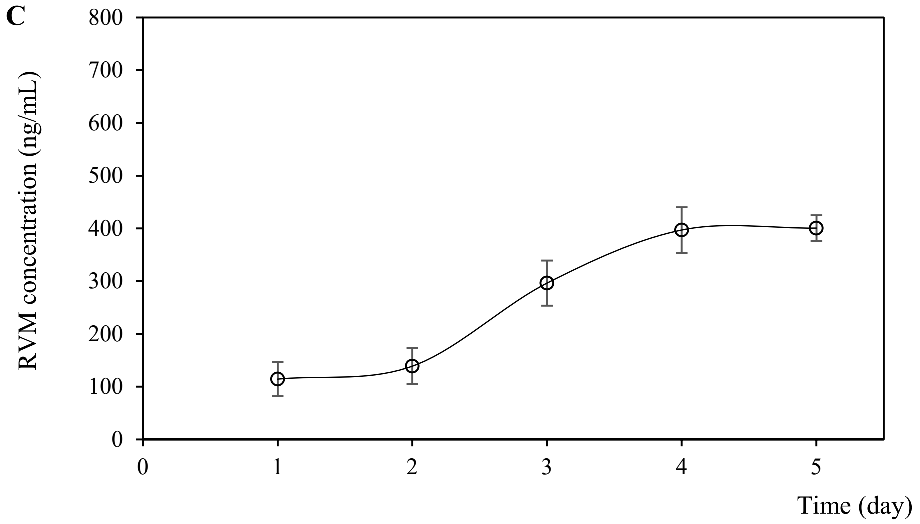
C
RVM concentration (ng/mL)
800
700
600
500
400
300
200
100
0
0
1
2
3
4
5
Time (day)

Figure S6. Kinetics profiles of rabies matrix protein (RVM) production (measured by the signal intensity of the dot blot membrane labeled with anti-RVM antibody) from Sf9 cells co-infected with recombinant baculoviruses carrying the rabies glycoprotein (BVG) and matrix protein (BVM) with a ratio between BVG and BVM of 2.5 using Schott flask as culture system. A) Coinfection 1: BVM 1 pfu/cell – BVG 2.5 pfu/cell. B) Coinfection 2: BVM 3 pfu/cell – BVG 7.5 pfu/cell. C) Coinfection 3: BVM 5 pfu/cell – BVG 12.5 pfu/cell. The points depict the average from three repetitions. Error bars represent the standard deviation from repetitions.

A

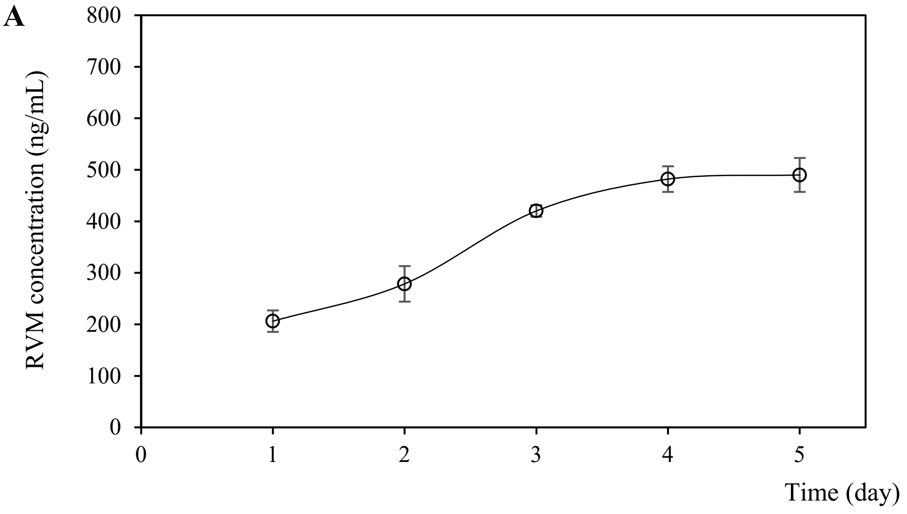

B

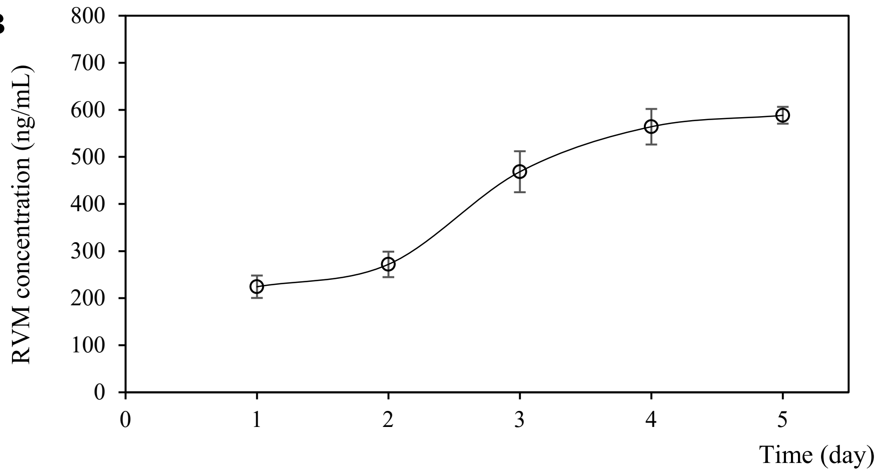

C

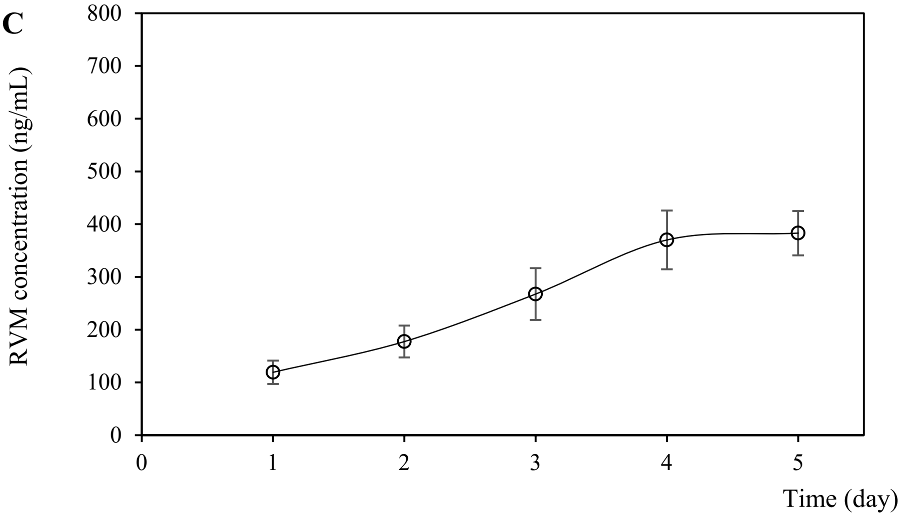

800
700
600
500
400
300
200
100
0
RVM concentration (ng/mL)
0
1
2
3
4
5
Time (day)

Figure S7. Perturbation graph of BVM MOI (A), BVG/BVM MOI ratio (B), and HT (C) on rabies M protein concentration (RVM) (determined by Dot-blot) modelled statistically.

A

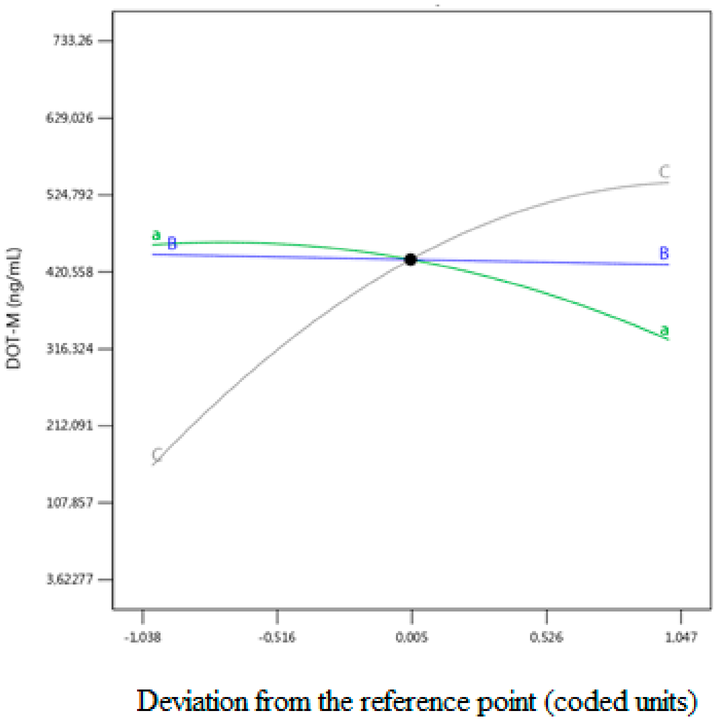

Figure S8. Kinetics profiles of rabies G glycoprotein (RVGP) production (measured by the signal intensity of the dot blot membrane labeled with anti-RVGP antibody) from Sf9 cells co-infected with recombinant baculoviruses carrying the rabies glycoprotein (BVG) and matrix protein (BVM) with a ratio between BVG and BVM of 1.5 using Schott flask as culture system. A) Coinfection 1: BVM 1 pfu/cell – BVG 1.5 pfu/cell. B) Coinfection 2: BVM 3 pfu/cell – BVG 4.5 pfu/cell. C) Coinfection 3: BVM 5 pfu/cell – BVG 7.5 pfu/cell. The points depict the average from three repetitions. Error bars represent the standard deviation from repetitions.

A

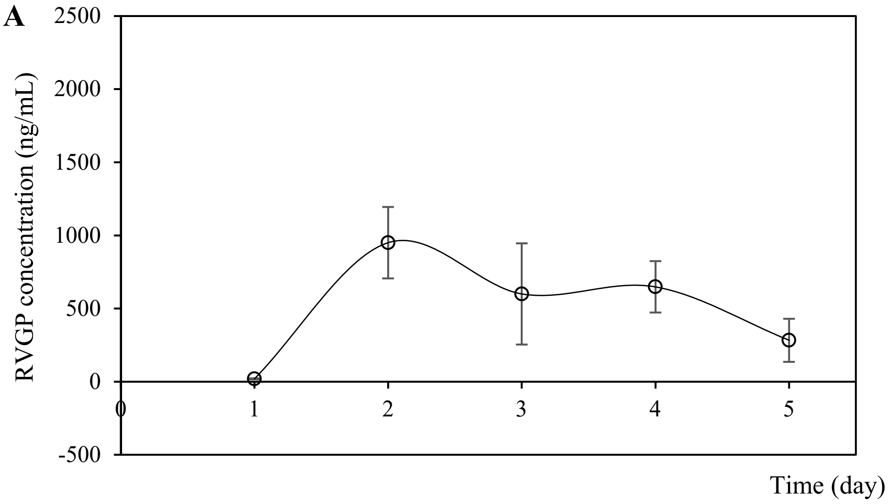

B

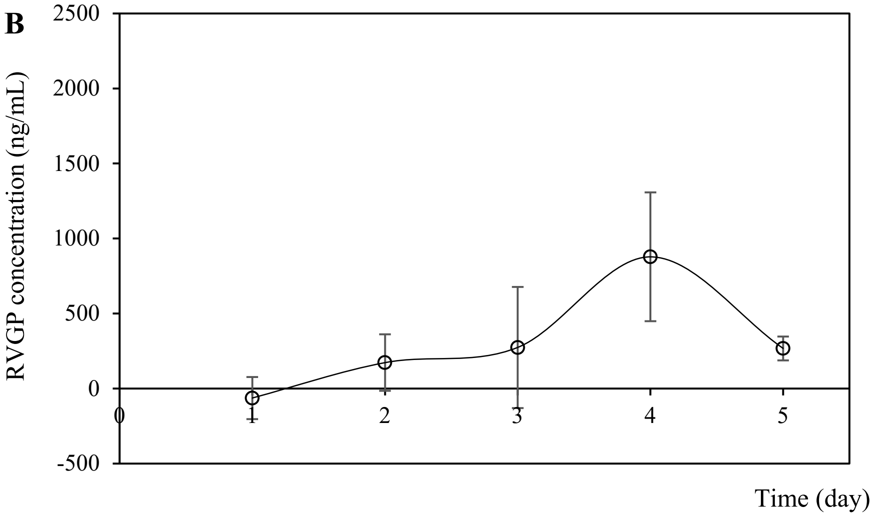

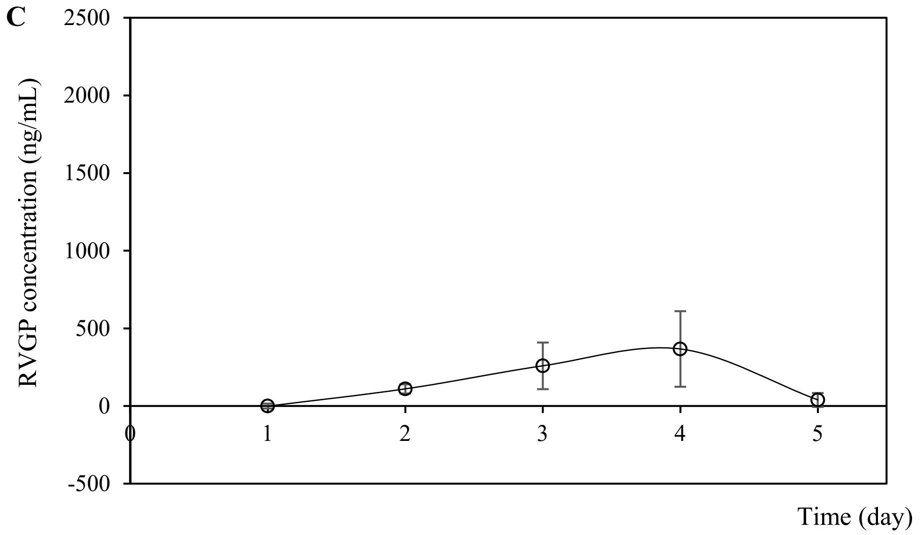
C
RVGP concentration (ng/mL)
2500
2000
1500
1000
500
0
-500
0
1
2
3
4
5
Time (day)

Figure S9. Kinetics profiles of rabies G glycoprotein (RVGP) production (measured by the signal intensity of the dot blot membrane labeled with anti-RVGP antibody) from Sf9 cells co-infected with recombinant baculoviruses carrying the rabies glycoprotein (BVG) and matrix protein (BVM) with a ratio between BVG and BVM of 2.0 using Schott flask as culture system. A) Coinfection 1: BVM 1 pfu/cell – BVG 2.0 pfu/cell. B) Coinfection 2: BVM 3 pfu/cell – BVG 6.0 pfu/cell. C) Coinfection 3: BVM 5 pfu/cell – BVG 10.0 pfu/cell. The points depict the average from three repetitions. Error bars represent the standard deviation from repetitions.

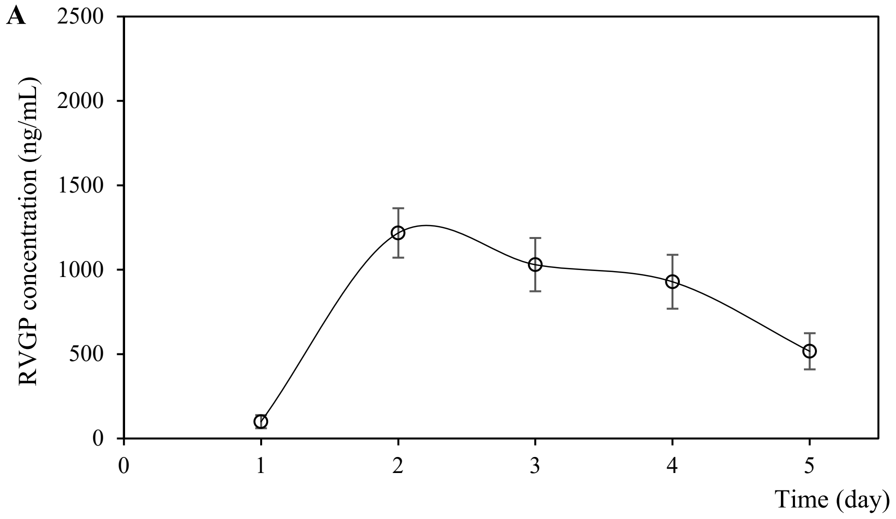

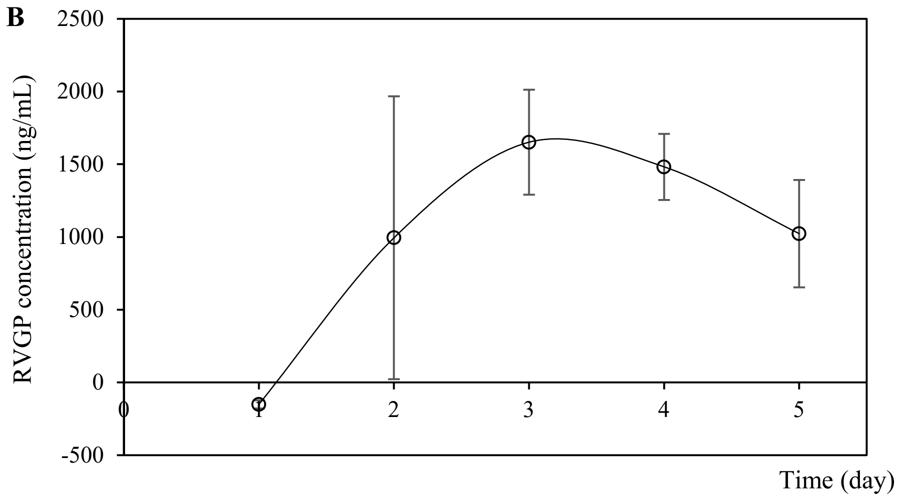

C

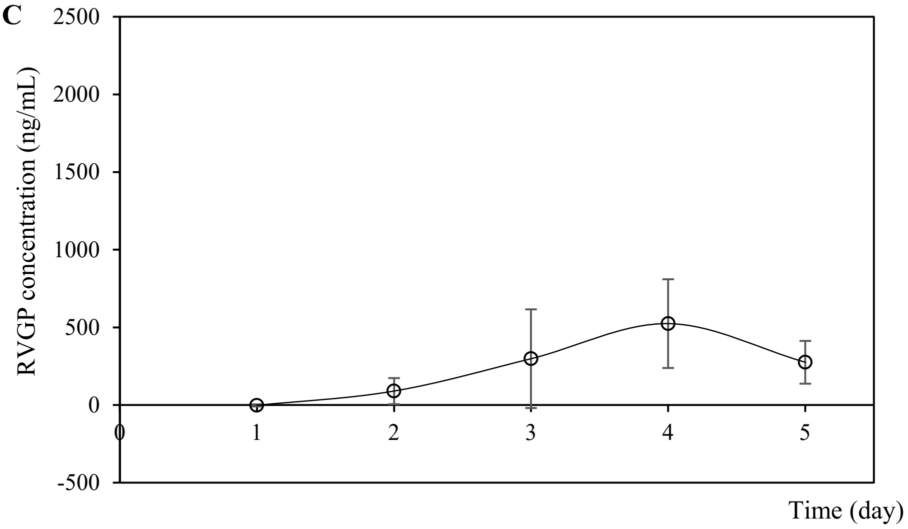

RVGP concentration (ng/mL)
2500
2000
1500
1000
500
0
-500
0
1
2
3
4
5
Time (day)

Figure S10. Kinetics profiles of rabies G glycoprotein (RVGP) production (measured by the signal intensity of the dot blot membrane labeled with anti-RVGP antibody) from Sf9 cells co-infected with recombinant baculoviruses carrying the rabies glycoprotein (BVG) and matrix protein (BVM) with a ratio between BVG and BVM of 2.5 using Schott flask as culture system. A) Coinfection 1: BVM 1 pfu/cell – BVG 2.5 pfu/cell. B) Coinfection 2: BVM 3 pfu/cell – BVG 7.5 pfu/cell. C) Coinfection 3: BVM 5 pfu/cell – BVG 12.5 pfu/cell. The points depict the average from three repetitions. Error bars represent the standard deviation from repetitions.

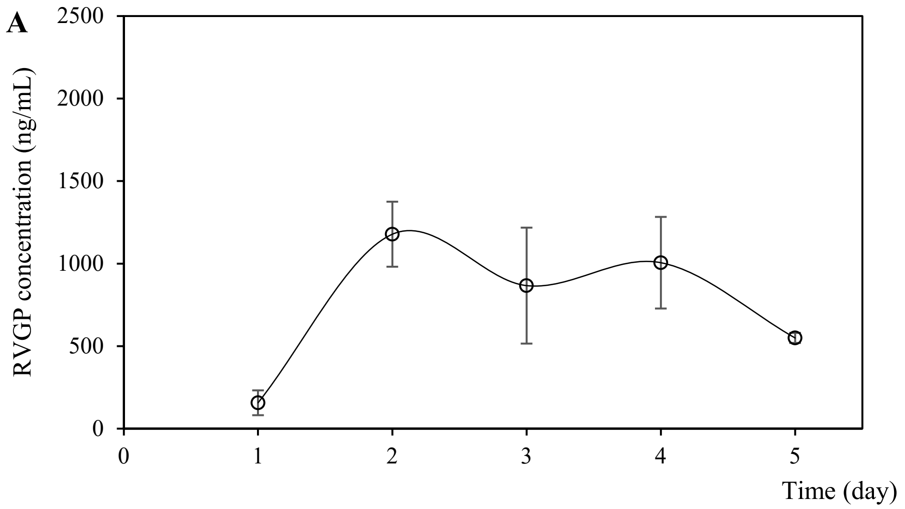

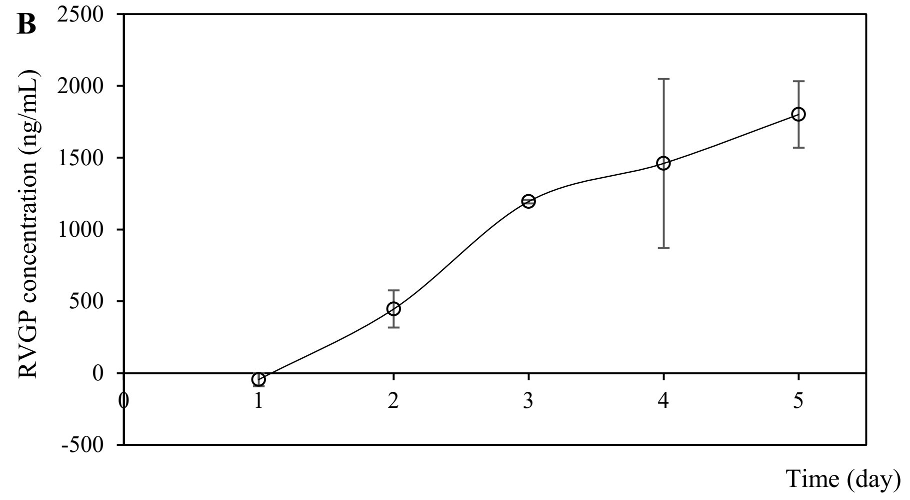

C

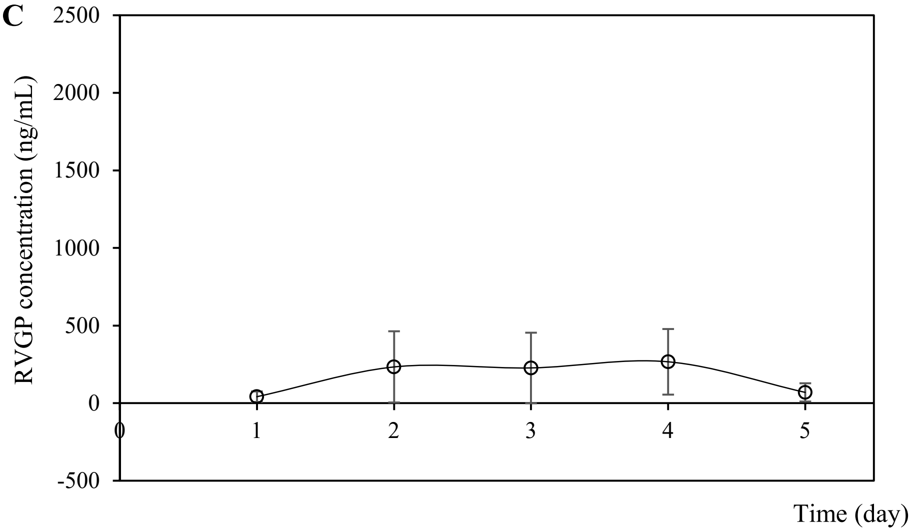

2500
2000
1500
1000
500
0
-500
RVGP concentration (ng/mL)
0
1
2
3
4
5
Time (day)

Figure S11. Perturbation graph of BVM MOI (A), BVG/BVM MOI ratio (B), and HT (C) on rabies G glycoprotein concentration (RVGP) (determined by Dot-blot) modelled statistically.

B

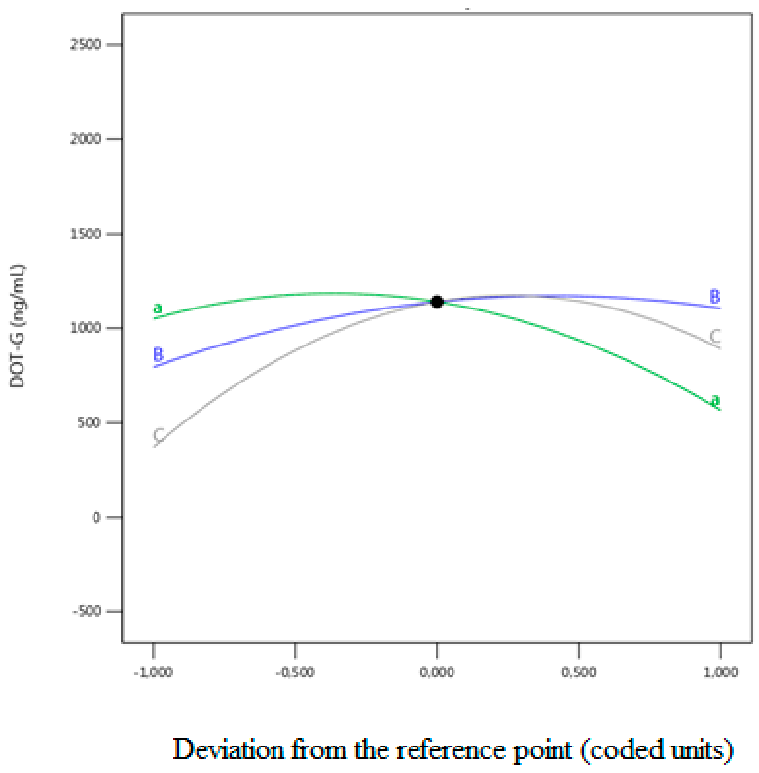

Figure S12. Kinetics profiles of rabies G glycoprotein (RVGP) production (measured by ELISA) from Sf9 cells co-infected with recombinant baculoviruses carrying the rabies glycoprotein (BVG) and matrix protein (BVM) with a ratio between BVG and BVM of 1.5 using Schott flask as culture system. A) Coinfection 1: BVM 1 pfu/cell – BVG 1.5 pfu/cell. B) Coinfection 2: BVM 3 pfu/cell – BVG 4.5 pfu/cell. C) Coinfection 3: BVM 5 pfu/cell – BVG 7.5 pfu/cell. The points depict the average from three repetitions. Error bars represent the standard deviation from repetitions.

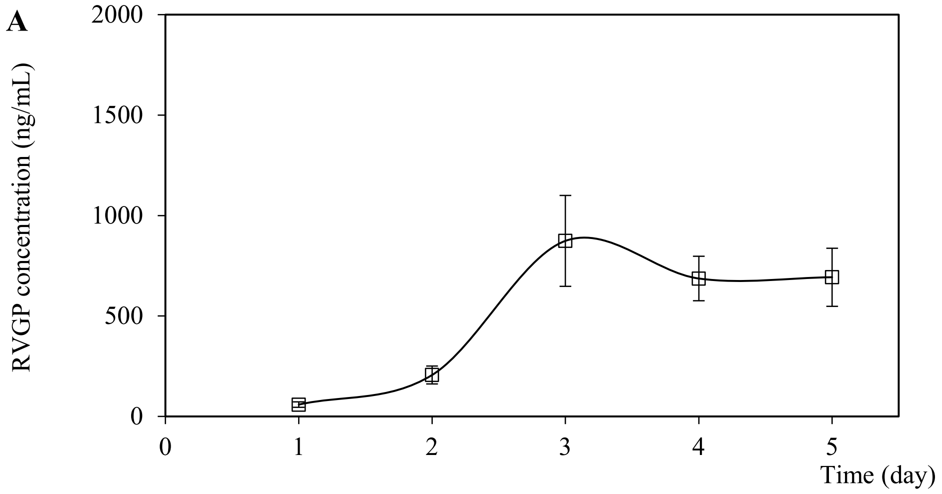

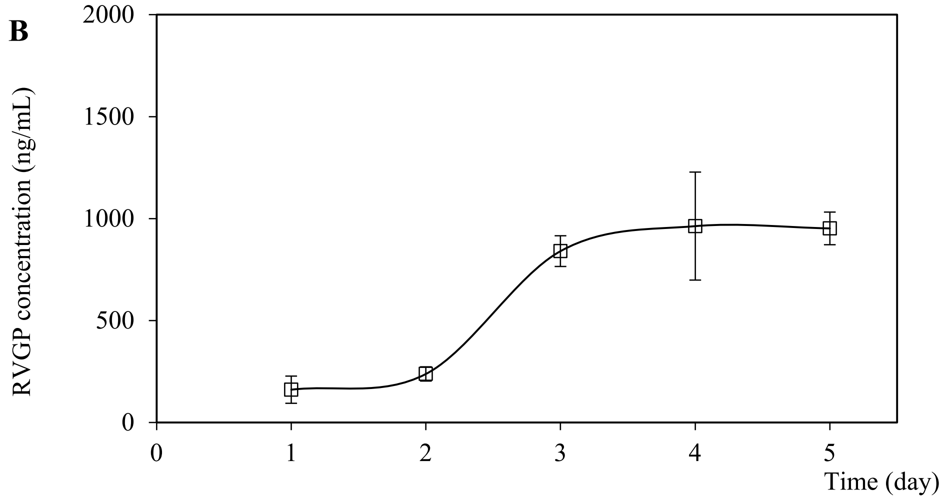

C

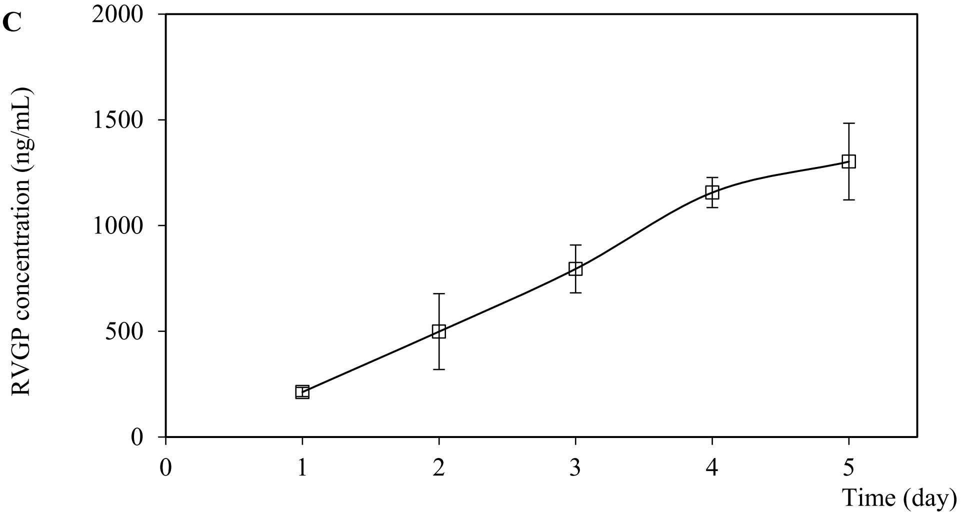

RVGP concentration (ng/mL)
2000
1500
1000
500
0
0
1
2
3
4
5
Time (day)

Figure S13. Kinetics profiles of rabies G glycoprotein (RVGP) production (measured by ELISA) from Sf9 cells co-infected with recombinant baculoviruses carrying the rabies glycoprotein (BVG) and matrix protein (BVM) with a ratio between BVG and BVM of 2.0 using Schott flask as culture system. A) Coinfection 1: BVM 1 pfu/cell – BVG 2.0 pfu/cell. B) Coinfection 2: BVM 3 pfu/cell – BVG 6.0 pfu/cell. C) Coinfection 3: BVM 5 pfu/cell – BVG 10.0 pfu/cell. The points depict the average from three repetitions. Error bars represent the standard deviation from repetitions.

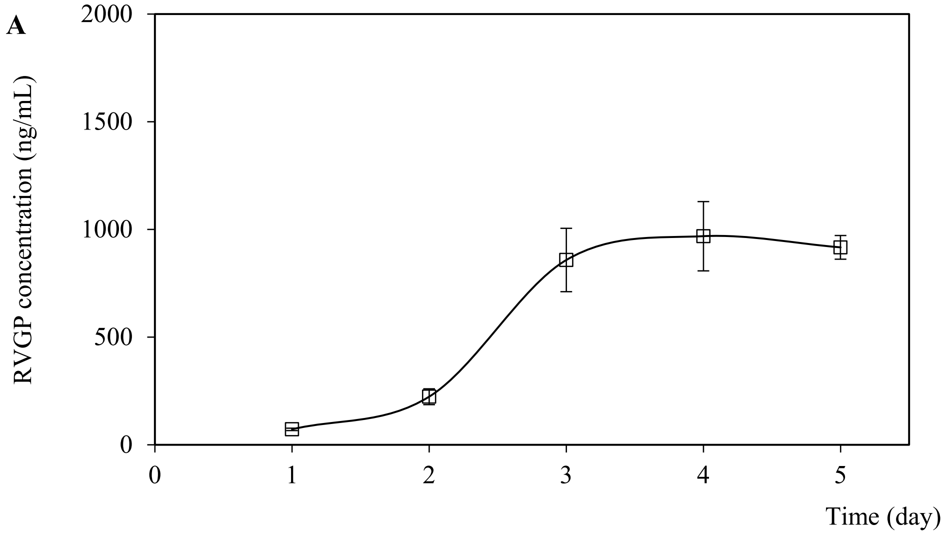

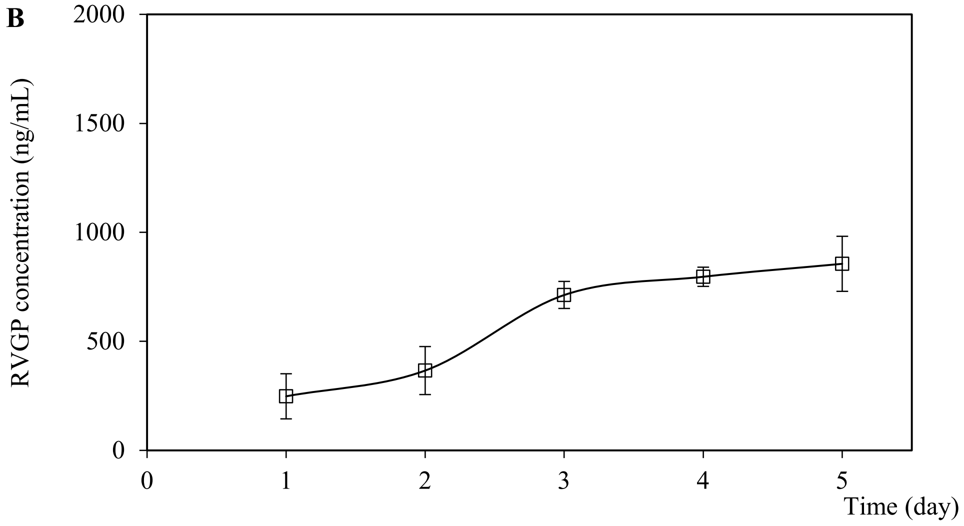

C

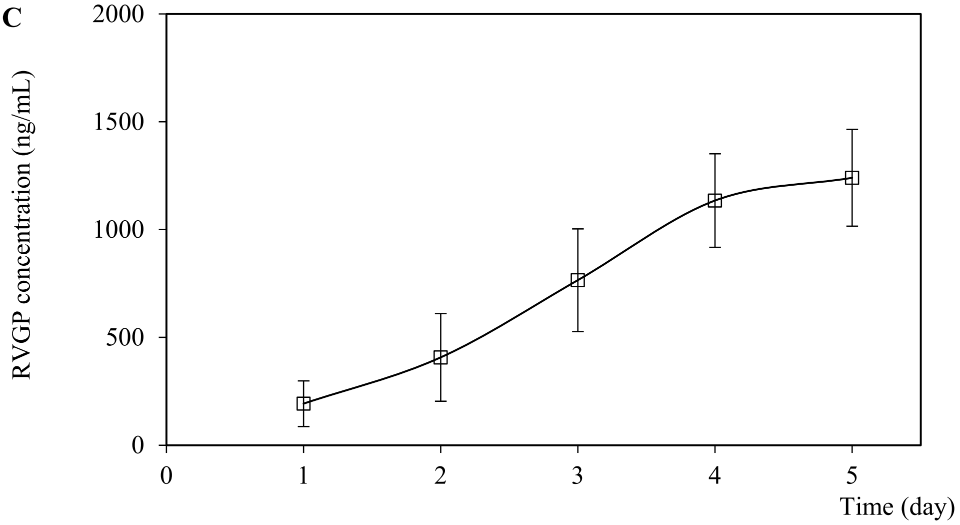
2000
1500
1000
500
0
RVGP concentration (ng/mL)
0
1
2
3
4
5
Time (day)

Figure S14. Kinetics profiles of rabies G glycoprotein (RVGP) production (measured by ELISA) from Sf9 cells co-infected with recombinant baculoviruses carrying the rabies glycoprotein (BVG) and matrix protein (BVM) with a ratio between BVG and BVM of 2.5 using Schott flask as culture system. A) Coinfection 1: BVM 1 pfu/cell – BVG 2.5 pfu/cell. B) Coinfection 2: BVM 3 pfu/cell – BVG 7.5 pfu/cell. C) Coinfection 3: BVM 5 pfu/cell – BVG 12.5 pfu/cell. The points depict the average from three repetitions. Error bars represent the standard deviation from repetitions.

A

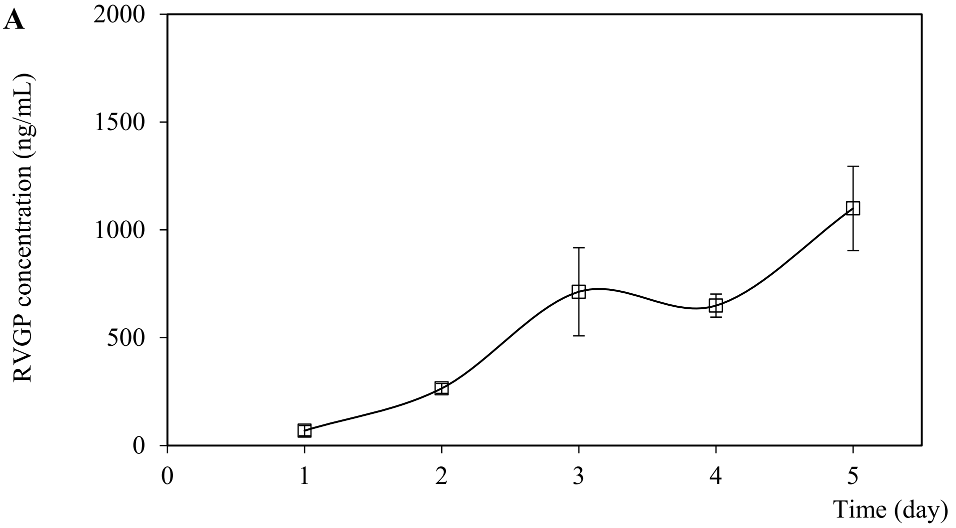

B

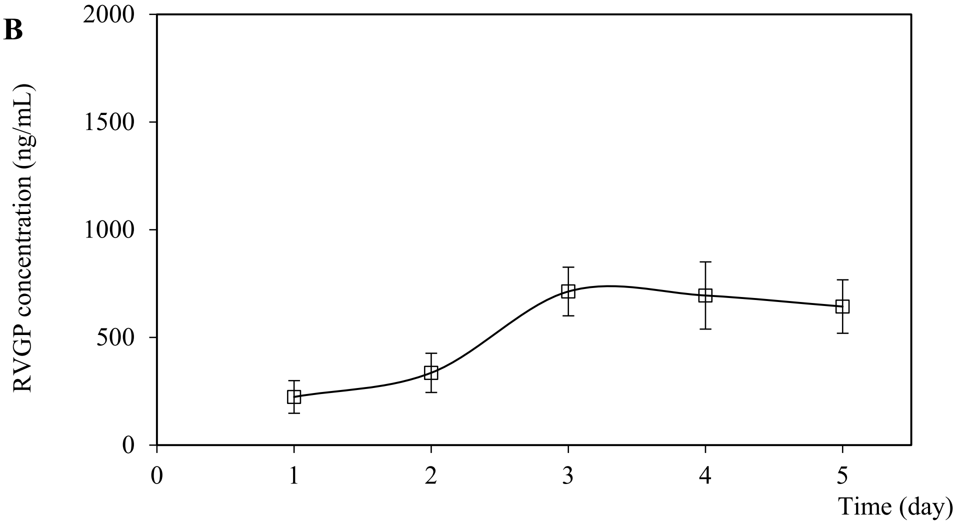

C

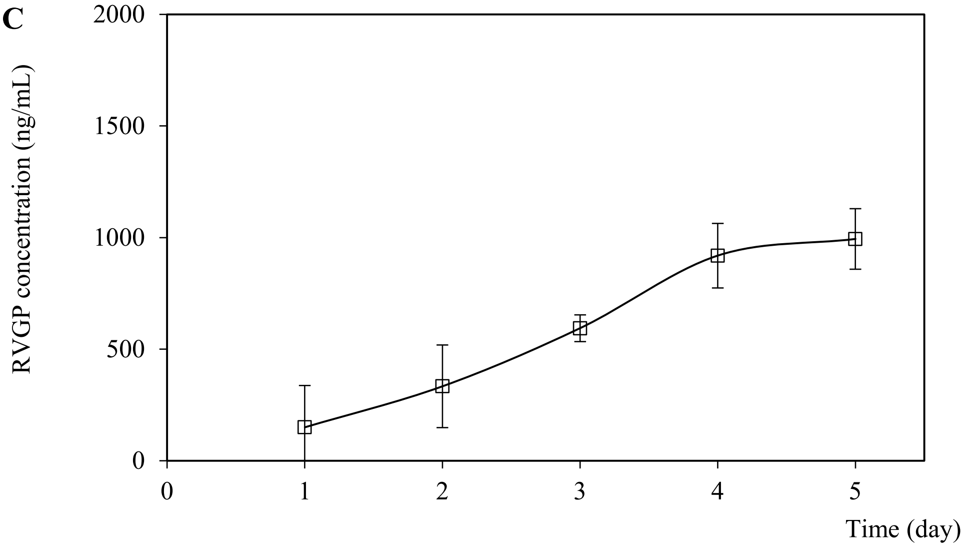
RVGP concentration (ng/mL)
2000
1500
1000
500
0
0
1
2
3
4
5
Time (day)

Figure S15. Perturbation graph of BVM MOI (A), BVG/BVM MOI ratio (B), and HT (C) on rabies G glycoprotein concentration (RVGP) (determined by ELISA) modelled statistically.

C

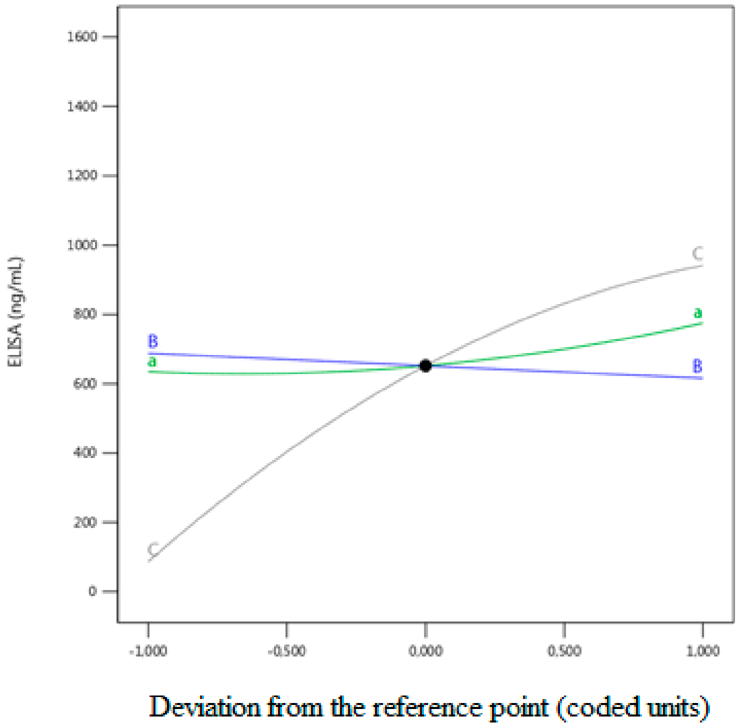

Supplement: Supplementary file 1 [file vaccines-11-00039-s001.zip › vaccines-1952315-supplementary.pdf]
